# Association of Long-term Exposure to Elevated Lipoprotein(a) Levels With Parental Life Span, Chronic Disease–Free Survival, and Mortality Risk: A Mendelian Randomization Analysis

**DOI:** 10.1001/jamanetworkopen.2020.0129

**Published:** 2020-02-28

**Authors:** Benoit J. Arsenault, William Pelletier, Yannick Kaiser, Nicolas Perrot, Christian Couture, Kay-Tee Khaw, Nicholas J. Wareham, Yohan Bossé, Philippe Pibarot, Erik S. G. Stroes, Patrick Mathieu, Sébastien Thériault, S. Matthijs Boekholdt

**Affiliations:** 1 Québec Heart and Lung Institute, Québec City, Québec, Canada; 2 Department of Medicine, Faculty of Medicine, Université Laval, Québec City, Québec, Canada; 3 Department of Cardiology, Amsterdam University Medical Center, University of Amsterdam, Amsterdam, the Netherlands; 4 MRC Epidemiology Unit, University of Cambridge, Cambridge, United Kingdom; 5 Department of Public Health and Primary Care, University of Cambridge, Cambridge, United Kingdom; 6 Department of Molecular Medicine, Faculty of Medicine, Université Laval, Québec City, Québec, Canada; 7 Department of Surgery, Faculty of Medicine, Université Laval, Québec City, Québec, Canada; 8 Department of Molecular Biology, Medical Biochemistry and Pathology, Faculty of Medicine, Université Laval, Québec City, Québec, Canada

## Abstract

**Importance:**

Elevated lipoprotein(a) (Lp[a]) levels are associated with atherosclerotic cardiovascular diseases. The association between high Lp(a) levels and human longevity phenotypes is, however, controversial.

**Objective:**

To examine whether genetically determined Lp(a) levels are associated with parental life span and chronic disease–free survival (health span) and the association between Lp(a) levels and long-term, all-cause mortality risk.

**Design, Setting, and Participants:**

In this genetic association study, cross-sectional mendelian randomization (UK Biobank [2006-2010] and LifeGen Consortium) and prospective analyses (European Prospective Investigation Into Cancer and Nutrition (EPIC)-Norfolk [1993-1997, with patients followed up to 2016]) were conducted using individual-level data on 139 362 participants. The association between a weighted genetic risk score of 26 independent single-nucleotide polymorphisms at the *LPA* locus on parental life span using individual participant data from the UK Biobank, as well as with summary statistics of a genome-wide association study of more than 1 million life spans (UK Biobank and LifeGen), were examined. The association between these single-nucleotide polymorphisms and the age at the end of the health span was tested using summary statistics of a previous genome-wide association study in the UK Biobank. The association between Lp(a) levels and all-cause mortality in the EPIC-Norfolk study was also investigated. Data were analyzed from December 2018 to December 2019.

**Exposures:**

Genetically determined and measured Lp(a) levels.

**Main Outcomes and Measures:**

Parental life span, health span, and all-cause mortality.

**Results:**

In 139 362 white British participants (mean [SD] age, 62.8 [3.9] years; 52% women) from the UK Biobank, increases in the genetic risk score (weighted for a 50-mg/dL increase in Lp[a] levels) were inversely associated with a high parental life span (odds ratio, 0.92; 95% CI, 0.89-0.94; *P* = 2.7 × 10^−8^). Using the Egger-mendelian randomization method, a negative association between *LPA* single-nucleotide polymorphisms and parental life span (mean [SD] Egger-mendelian randomization slope, −0.0019 [0.0002]; *P* = 2.22 × 10^−18^) and health span (−0.0019 [0.0003]; *P* = 3.00 × 10^−13^) was noted. In 18 720 participants from EPIC-Norfolk (5686 cases), the mortality risk for those with Lp(a) levels equal to or above the 95th percentile was equivalent to being 1.5 years older in chronologic age (β coefficient [SE], 0.194 [0.064]).

**Conclusions and Relevance:**

The results of this study suggest a potential causal effect of absolute Lp(a) levels on human longevity as defined by parental life span, health span, and all-cause mortality. The results also provide a rationale for trials of Lp(a)-lowering therapy in individuals with high Lp(a) levels.

## Key Points

**Question:**

Is long-term exposure to elevated lipoprotein(a) levels associated with shorter life span?

**Findings:**

In this genetic association study including 139 362 participants, 2-sample mendelian randomization showed that genetically elevated lipoprotein(a) levels were associated with parental life span. Measured lipoprotein(a) levels were also associated with all-cause mortality in a population-based study.

**Meaning:**

Results of this study provide additional knowledge on the potential biological determinants of human longevity phenotypes and a rationale for trials of lipoprotein(a)-lowering therapy in individuals with high lipoprotein(a) levels.

## Teaser

This genetic association study uses mendelian randomization to examine the association between genetically determined lipoprotein(a) levels and the parental life span, chronic disease–free survival, and mortality risk of individuals with elevated levels.

## Introduction

Lipoprotein(a) (Lp[a]) consists of a low-density lipoprotein attached to apolipoprotein(a) by a disulfide bond. Plasma levels of Lp(a) are associated with a higher risk of a broad range of atherosclerotic cardiovascular disease (CVD).^1,2,3,4,5^ The evidence linking Lp(a) levels and Lp(a)-raising genetic variants with all-cause mortality is not as consistent. A 1998 study of healthy centenarian individuals initiated a debate about the potential association between Lp(a) and longevity following the report that up to one-quarter of that population had high Lp(a) levels in the absence of any atherosclerotic CVD.^6^ Another study of patients with documented coronary heart disease found no evidence of an association between high Lp(a) levels and all-cause mortality.^7^ A recently published study by Langsted et al^8^ revealed an association between high Lp(a) levels and cardiovascular and all-cause mortality in the general Danish population. This association could be owing to the fact that individuals with high Lp(a) levels are typically characterized by a smaller apolipoprotein(a) isoform size.

Whether high Lp(a) levels predict human longevity phenotypes is an issue of particular relevance as Lp(a)-lowering therapies are currently being developed; one of them (an antisense oligonucleotide against *LPA* called AKCEA-APO[a]-L_rx_)^9^ is expected to be tested in a planned large, phase 3 cardiovascular outcomes trial. Determining the association between high Lp(a) levels in large, prospective studies would provide information on the potential of these therapies to extend the life span in individuals with high Lp(a) levels.

The definition of what constitutes longevity in human genetic studies is highly debated, and the lack of a universally recognized definition increases the possibility of biases, hindering external validation efforts, especially for case-control studies.^10^ Results of many studies on centenarian or other long-lived individuals might have been confounded by the use of different birth cohorts of centenarians and controls, selection bias, or survival bias. Parental life span is a novel and innovative tool that is increasingly used to study the genetic makeup of human longevity that considerably reduces selection bias as both cases and controls are uniformly recruited. Two genome-wide association studies identified variants at the *LPA* locus to be associated with shorter life span as estimated by parental life span.^11,12^

Although studying the genetic determinants of life span is necessary to improve our understanding of the complexity of human longevity, addressing the global challenges of aging is equally important to improve the quality of care of aging individuals. The association between measured and genetically determined Lp(a) levels and human longevity is controversial and, despite evidence suggesting that *LPA* might be a locus influencing longevity, it is unknown whether a concentration-dependent effect of Lp(a) levels on human longevity exists. In this study, we used a 2-sample mendelian randomization (MR) design to determine whether genetic variants associated with elevated Lp(a) levels are associated with human longevity phenotypes, as estimated by parental life span and the age at the end of the chronic disease–free survival (health span), in the UK Biobank. We also investigated the association between measured and genetically determined Lp(a) levels and long-term all-cause and cardiovascular mortality in another cohort from the United Kingdom: the European Prospective Investigation Into Cancer and Nutrition (EPIC)-Norfolk study.

## Methods

### Study Populations

We used a 2-sample cross-sectional MR study design to assess the relationship between genetically predicted Lp(a) levels and longevity phenotypes. eFigure 1 in the Supplement presents further details on the design of the study and a description of how exposures and outcomes were defined. The association between *LPA* variants and parental life span and the age at the end of the health span was assessed in the UK Biobank. Our MR analysis included 139 362 white individuals between ages 55 and 69 years recruited between 2006 and 2010 in several centers in the United Kingdom (eMethods 1 in the Supplement).^13^ Data analysis was conducted between December 2018 and December 2019. The association between genetically determined and measured Lp(a) levels and long-term all-cause and cardiovascular mortality was assessed in the EPIC-Norfolk study, which is a population-based study of 25 663 men and women aged 45 to 79 years residing in Norfolk, United Kingdom. Participants were recruited by mail from age-sex registers of general practices in Norfolk. The design, methods of the study, and baseline characteristics of the study participants have been described previously.^4,14^ At the baseline survey conducted between 1993 and 1997 (with patients followed up to 2016), participants completed a detailed health and lifestyle questionnaire. Lipoprotein(a) levels were measured with an immune-turbidimetric assay using polyclonal antibodies directed against epitopes in apolipoprotein(a) (Denka Seiken), as previously described.^15^ The distribution of Lp(a) in participants of EPIC-Norfolk is presented in eFigure 2 in the Supplement. The Norwich District Health Authority Ethics Committee approved the study, and all participants gave signed informed consent; no financial compensation was provided. This report followed the Strengthening the Reporting of Genetic Association Studies (STREGA) reporting guideline whenever possible (summary statistics were used in most analyses, and individual participant data were not always available).

### Outcomes Ascertainment and Definitions

Weighted genetic risk scores (wGRSs) for Lp(a) levels were engineered using data from the studies of Burgess et al^16^ and Mack et al^17^ and are described in eMethods 2 in the Supplement. A recent analysis suggested that the association between genetically determined Lp(a) levels and cardiovascular outcomes could be heterogeneous across studies that have used different instruments and different assays to measure Lp(a) levels.^18^ Participants were asked the current age of their parents or the age at which their parents had died. We used the definition of Pilling et al^19^ (described in eMethods 1 in the Supplement and eFigure 3 in the Supplement) to define high parental life span in participants of the UK Biobank. We also used summary statistics from a genome-wide association study of Timmers et al^11^ (joint analysis of the UK Biobank and the LifeGen Consortium) and another one by Zenin et al^20^ (age at the end of the health span in the UK Biobank) to study the association between *LPA* variants and parental life span. In the combined analysis of the UK Biobank and LifeGen Consortium (26 additional population cohorts), the genetic architecture of human longevity was studied using more than 1 million parental life spans with Cox proportional hazards regression models, as previously described.^11^ To identify genetic loci associated with human health span, Zenin et al^20^ performed a genome-wide association study on disease-free survival (age at the first occurrence of a major chronic disease, including cancer, diabetes, congestive heart failure, myocardial infarction, chronic obstructive pulmonary disease, stroke, dementia, or death) using the Cox-Gompertz proportional hazards regression model.

In EPIC-Norfolk, all individuals were flagged for mortality at the UK Office of National Statistics, with vital status ascertained for the entire cohort. Death certificates for all decedents were coded by trained nosologists according to the *International Classification of Diseases, Ninth Revision*. In addition, participants admitted to the hospital were identified by their unique National Health Service number by data linkage with the East Norfolk Health Authority database, which identifies all hospital contacts throughout England and Wales for Norfolk residents. In EPIC-Norfolk among 18 720 individuals with Lp(a) measurement, 5686 died (2412 of CVD) during the follow-up. Additional details on genotyping and selection of genetic instruments are described in eMethods 2 in the Supplement.

### Statistical Analysis

To evaluate the association between genetically determined Lp(a) levels and parental life span in the UK Biobank, we performed 2-sample MR, in which the association between the selected single-nucleotide polymorphisms (SNPs) and Lp(a) levels were obtained from Burgess et al^16^ and the association of the SNPs with parental life span was assessed in the UK Biobank. First, we separated individuals in the UK Biobank into quartiles based on wGRS distribution and performed logistic regression, adjusting for age, sex, and the first 10 ancestry-based principal components to document the association between genetically elevated Lp(a) levels and parental life span. Second, we obtained effect estimates (adjusted for the minor allele frequency of each variant) by a 50-mg/dL increase in Lp(a) levels, a threshold recently reported by Langsted et al.^8^ We used inverse-variance-weighted MR (IVW-MR) and performed a meta-analysis of each Wald ratio (the effect of the genetic instrument on Lp[a] levels divided by its effect on parental life span). To determine the significance of the associations, a bootstrap method was used. A 2-tailed *P* value was calculated using a *z* test from 100 000 random simulations. The IVW-MR is considered one of the simplest ways to obtain MR estimates using multiple SNPs. The limitation of IVW-MR is the assumption that SNPs do not have pleiotropic effects (effects on variables other than the trait of interest). To determine the presence of unmeasured pleiotropy, we performed Egger-MR in which a nonzero y intercept is allowed to assess violation of IVW-MR as described by Bowden et al.^21^ These analyses were performed using R, version 3.5.1 (R Foundation). In the IVW-MR analyses, all *P* values <.0083 (0.05/6 outcomes) were considered as statistically significant.

In EPIC-Norfolk, Cox proportional hazards regression models were used to calculate hazard ratios (HRs) and corresponding 95% CIs for the risk of all-cause and cardiovascular mortality associated with various thresholds of measured Lp(a) levels and 2 SNPs associated with high Lp(a) levels. Hazard ratios for all-cause and cardiovascular mortality were obtained before and after adjusting for cardiovascular risk factors (age, sex, smoking, body mass index, systolic blood pressure, diabetes, and creatinine level when evaluating measured Lp[a] levels and age and sex when evaluating Lp[a]-increasing SNPs). We estimated the difference in survival between those with high (≥95th percentile) vs low (<50th percentile) Lp(a) levels in age-equivalent terms by dividing the β coefficient for all-cause mortality associated with high vs low Lp(a) levels by the β coefficient difference in all-cause mortality associated with 1-year increases in age, as previously described.^22,23^ In EPIC-Norfolk, we also investigated the association between 2 SNPs with a strong association with Lp(a) levels (rs10455872 and rs3798220) and all-cause and cardiovascular mortality. These analyses were performed using SPSS software, version 12.0.1 (IBM SPSS). In the prospective analyses, all 2-tailed *P* values <.05 were considered as statistically significant.

## Results

**Figure 1.**
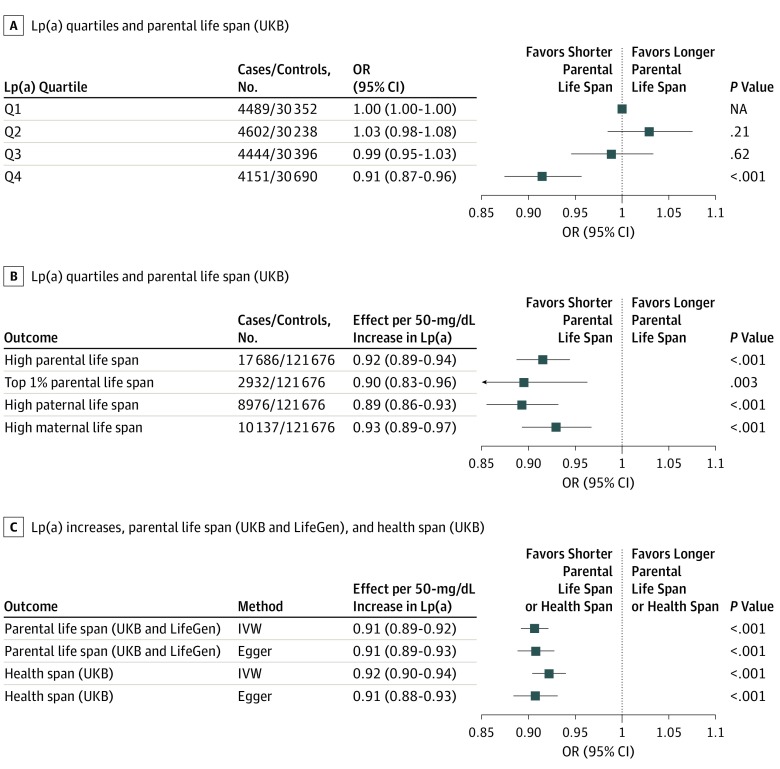
Association Between the Lp(a) Genetic Instruments, Parental Life Span, and Health Span A, High parental life span in participants of the UK Biobank (UKB) separated into quartiles of the *Lp(a) *weighted genetic risk score (wGRS) from Burgess et al.^16^ B, High parental life span, top 1% parental life span, high paternal life span, and high maternal life span associated with a 50-mg/dL increase in the *LPA* wGRS in the UK Biobank from Burgess et al.^16^ C, Parental life span and age at the end of the health span. Models were adjusted for age, sex, and the 10 first ancestry-based principal components. IVW indicates inverse-variance weighted; Lp(a), lipoprotein(a); NA, not applicable; OR, odds ratio; and Q, quartile. Error bars indicate 95% CIs.

Of the 139 362 UK Biobank participants included in this analysis (mean [SD] age, 62.8 [3.9] years; 52% women), 17 686 were considered as having high parental life span (at least 1 long-lived parent; father still alive and age >90 y or father’s age at death ≥90 y, or mother still alive and >93 y or mother’s age of death ≥93 y), and 2932 individuals were defined as having 1 parent with exceptional longevity (top 1% survival). The definition of parental life span phenotypes is described in eMethods 1 in the Supplement. In the sex-specific analyses investigating paternal and maternal survival, 8976 individuals were considered as having high paternal life span and 10 137 were considered as having high maternal life span. Regardless of how longevity was defined and across all wGRSs used to weight Lp(a) levels, genetically determined Lp(a) (whether examined as quartiles of the wGRS or as continuous GRS) was negatively associated with a high parental life span in the UK Biobank. The odds ratios for a high parental life span in the UK Biobank study population separated into quartiles of the Lp(a) wGRS are presented in Figure 1A and eFigure 4 in the Supplement (HR, 0.91; 95% CI, 0.87-0.96). Genetically determined Lp(a) levels were negatively associated with parental life span and paternal and maternal life span separately. The association per 50-mg/dL increase in Lp(a) (odds ratio, 0.92; 95% CI, 0.89-0.94; *P* = 2.7 × 10^−8^) is presented in Figure 1B and eFigure 4 in the Supplement (HR, 0.92; 95% CI, 0.89-0.94). Figure 1C and eFigure 4 in the Supplement present the negative association between genetically determined Lp(a) levels and parental life span in the meta-analysis of the UK Biobank and LifeGen Consortium and at the end of health span in the UK Biobank.

**Figure 2.**
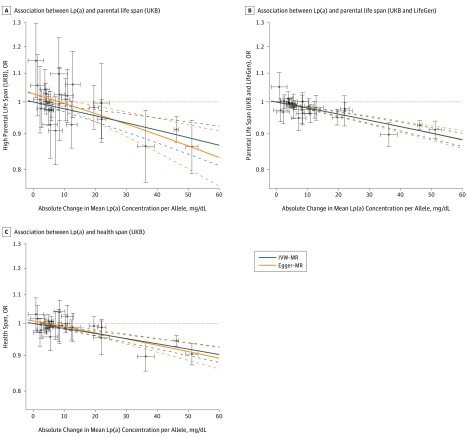
Mendelian Randomization Analysis of the Association Between Lipoprotein(a) (Lp[a]) and Longevity Phenotypes Association between single-nucleotide polymorphisms at the *LPA* locus weighted for their association with Lp(a) levels from the study of Burgess et al^16^ and higher parental ife span in the UK Biobank (UKB) (A), parental life span in the UKB and LifeGen meta-analysis (B), and age at the end of the health span (C). Each plotted point represents the association of a single genetic variant with Lp(a) levels and a high parental life span. The blue line represents the regression slope using the inverse-variance–weighted (IVW) method and the orange line represents the regression slope using the Egger method. Dashed lines indicate 95% CIs. MR indicates mendelian randomization; OR, odds ratio. Error bars indicate 95% CI.

Figure 2 presents the association between the 26 *LPA* SNPs with Lp(a) levels and high parental life span in the UK Biobank (Figure 2A), the meta-analysis of the UK Biobank and LifeGen Consortium (Figure 2B), and the age at the end of the health span (Figure 2C). We obtained estimates of causal effects of Lp(a) levels on parental life span in the UK Biobank using IVW-MR and Egger-MR (mean [SD] Egger-MR slope, −0.0019 [0.0002]; *P* = 2.22 × 10^−18^) and health span (−0.0019 [0.0003]; *P* = 3.00 × 10^−13^). Egger-MR analysis revealed no evidence of horizontal pleiotropy in the 2 outcomes that combined paternal and maternal life span (Table 1). There was, however, evidence of horizontal pleiotropy when maternal life span only was investigated (*P* value of intercept = .04). There was also no evidence of horizontal pleiotropy in the association between *LPA* SNPs and parental life span in the meta-analysis of the UK Biobank and the age at the end of the health span. Results presented in Figure 1 and Figure 2 were obtained using a wGRS, with genetic instruments and weights obtained from the study of Burgess et al.^16^ eFigures 4 and 5 in the Supplement present a technical replication of these findings using genetic instruments and weights on Lp(a) and Lp(a)-adjusted for apolipoprotein(a) isoform size obtained from the study of Mack et al.^17^ The association of each SNP from the study of Burgess et al^16^ and Mack et al^17^ with these same outcomes are presented in eFigures 6, 7, and 8 and in eTables 2, 3, and 4 in the Supplement.

**Table 1.** Estimates of the Association Between Lipoprotein(a) Levels and Parental Life Span in the UKB and LifeGen Consortium

Outcome	IVW-MR, Slope Estimate (SD)	*P* Value	Egger-MR, Slope Estimate (SD)	*P* Value	Intercept	*P* Value^a^
High parental life span (UKB)	−0.0020 (0.0004)	3.17 × 10^−9^	−0.0026 (0.0004)	1.68 × 10^−8^	0.0038	.08
Top 1% parental life span (UKB)	−0.0027 (0.0008)	1.97 × 10^−5^	−0.0020 (0.0011)	.06	−0.0048	−.35
High paternal life span (UKB)	−0.0027 (0.0005)	1.43 × 10^−8^	−0.0028 (0.0005)	1.18 × 10^−5^	0.0006	.85
High maternal life span (UKB)	−0.0017 (0.0004)	1.60 × 10^−4^	−0.0025 (0.0006)	4.00 × 10^−5^	0.0056	.04
Parental life span (UKB and LifeGen)	−0.0020 (0.0002)	1.80 × 10^−32^	−0.0019 (0.0002)	2.22 × 10^−18^	−0.0007	.86
Health span (UKB)	−0.0016 (0.0002)	3.21 × 10^−16^	−0.0019 (0.0003)	3.00 × 10^−13^	0.0084	.09

Abbreviations: IVW, inverse-variance weighted; MR, mendelian randomization; UKB, UK Biobank.

^a^ A *P* < .05 indicates that the y-intercept of the MR regression line is significantly different from 0, suggesting unbalanced pleiotropy.

The baseline characteristics of the EPIC-Norfolk study participants by Lp(a) levels are presented in eTable 1 in the Supplement. Participants in the EPIC-Norfolk study were followed up for a mean of 20 years. Compared with participants with Lp(a) levels lower than 50 mg/dL, those with Lp(a) levels 50 mg/dL or higher had an increased HR of both all-cause and cardiovascular mortality (all-cause: HR, 1.17; 95% CI, 1.08-1.27; cardiovascular: HR, 1.54; 95% CI, 1.37-1.72) (Table 2). In sex-specific analyses, the association of high Lp(a) levels with cardiovascular mortality was observed in both men and women, while the association of high Lp(a) levels with all-cause mortality was statistically significant only in men. No associations were found with the risk of noncardiovascular mortality in the entire group and in the sex-specific analyses (eFigure 10 in the Supplement).

**Table 2.** Health Hazards Associated With Elevated Lipoprotein(a) Levels

Outcome^a^	All Participants		Men		Women	
	<50 mg/dL	≥50 mg/dL	<50 mg/dL	≥50 mg/dL	<50 mg/dL	≥50 mg/dL
All-cause mortality						
Cases/controls, event rate, No./No. (%)	4945/16 594 (29.8)	741/2126 (34.9)	2678/7504 (35.7)	359/879 (40.8)	2267/9090 (24.9)	382/1247 (30.6)
Model 1, HR (95% CI)	1 [Reference]	1.17 (1.08-1.27)	1 [Reference]	1.26 (1.13-1.40)	1 [Reference]	1.10 (0.99-1.23)
Model 2, HR (95% CI)	1 [Reference]	1.17 (1.08-1.27)	1 [Reference]	1.26 (1.13-1.41)	1 [Reference]	1.09 (0.98-1.22)
Cardiovascular mortality						
Cases/controls, event rate, No./No. (%)	2026/16 594 (12.2)	386/2126 (18.2)	1170/7504 (15.6)	208/879 (23.7)	856/9090 (9.4)	178/1247 (14.3)
Model 1, HR (95% CI)	1 [Reference]	1.52 (1.36-1.70)	1 [Reference]	1.70 (1.47-1.97)	1 [Reference]	1.33 (1.13-1.57)
Model 2, HR (95% CI)	1 [Reference]	1.54 (1.37-1.72)	1 [Reference]	1.77 (1.52-2.05)	1 [Reference]	1.32 (1.11-1.55)

Abbreviation: HR, hazard ratio.

^a^ Model 1 was adjusted for age and sex. Model 2 was adjusted for age, sex, smoking, body mass index, systolic blood pressure, diabetes, and estimated glomerular filtration rate.

Table 3 presents the association of Lp(a) with all-cause and cardiovascular mortality in participants above the 50th percentile of the Lp(a) level distribution. The risks for all-cause and cardiovascular mortality were highest in participants with Lp(a) levels equal to or above the 95th percentile (all-cause: HR, 1.17; 95% CI, 1.08-1.25; cardiovascular: HR, 1.54; 95% CI, 1.37-1.72). The association between Lp(a) levels and mortality causes by baseline age are presented in eFigure 9 in the Supplement. From the Cox proportional hazards regression model, the β coefficient (SE) for all-cause mortality associated with each year increase in chronologic age was 0.127 (0.003). The β coefficient (SE) for a comparison between high (≥95th percentile) vs low Lp(a) (<50th percentile) levels was 0.194 (0.064), which is equivalent to approximately 1.5 years in chronologic age for all-cause mortality risk. This analysis suggests that the mortality risk for individuals with Lp(a) levels equal to or above the 95th percentile is equivalent to being 1.5 years older in chronologic age.

**Table 3.** Health Hazards Associated With Very High Lipoprotein(a) Levels

Outcome^a^	Lipoprotein(a) Percentiles				
	<50	50-80	81-90	91-95	>95-100
Lipoprotein(a) range, mg/dL	<11.4	11.4 to <35.0	35.0 to <53.3	53.3 to <69.7	≥69.7
All-cause mortality					
Cases/controls, event rate (%)	2710/9365 (28.9)	1742/5614 (31.0)	568/1869 (30.4)	315/937 (33.6)	351/935 (37.5)
Model 1, HR (95% CI)	1 [Reference]	0.95 (0.89-1.01)	1.03 (0.94-1.13)	1.13 (1.00-1.27)	1.23 (1.10-1.38)
Model 2, HR (95% CI)	1 [Reference]	0.94 (0.89-1.00)	1.06 (0.97-1.16)	1.13 (1.00-1.27)	1.22 (1.09-1.37)
Cardiovascular mortality					
Cases/controls, event rate (%)	1062/9365 (11.3)	738/5614 (13.1)	260/1869 (13.9)	165/937 (17.6)	187/935 (20.0)
Model 1, HR (95% CI)	1 [Reference]	1.01 (0.92-1.11)	1.21 (1.06-1.38)	1.54 (1.30-1.81)	1.70 (1.45-1.98)
Model 2, HR (95% CI)	1 [Reference]	1.00 (0.91-1.10)	1.26 (1.10-1.44)	1.52 (1.29-1.80)	1.71 (1.46-2.00)

Abbreviation: HR, hazard ratio.

^a^ Model 1 was adjusted for age and sex. Model 2 was adjusted for age, sex, smoking, body mass index, systolic blood pressure, diabetes, and estimated glomerular filtration rate.

For rs10455872, compared with noncarriers (AA genotype; event rate of 29.6% for all-cause mortality and 12.2% for cardiovascular mortality), those who carried at least 1 Lp(a)-raising allele (AG or GG genotype) were at higher risk for all-cause (HR, 1.14; 95% CI, 1.07-1.22; event rate, 31.9%) and cardiovascular (HR, 1.23; 95% CI, 1.11-1.36; event rate, 13.9%) mortality. For rs3798220, compared with noncarriers (TT genotype; event rate, 29.9% for all-cause mortality and 12.4% for cardiovascular mortality), those who carried at least 1 Lp(a)-raising allele (TC or CC genotype) were, however, not at significantly higher risk for all-cause (HR, 1.03; 95% CI, 0.90-1.18; event rate, 29.3%) and cardiovascular (HR, 1.16; 95% CI, 0.94-1.42; event rate, 13.4%) mortality. Compared with individuals without an Lp(a)-raising allele, those with only 1 Lp(a)-raising allele (in rs10455872 or rs3798220) had an increased risk of both all-cause and cardiovascular mortality (eFigure 11 in the Supplement). Those with 2 or more Lp(a)-raising alleles had an even higher risk of all-cause and cardiovascular mortality, although the association with cardiovascular mortality did not reach statistical significance (HR, 1.40; 95% CI, 0.98-2.00). However, there were only 202 individuals in that subcategory, including 30 who died of CVD. No associations were found with the risk of noncardiovascular mortality.

## Discussion

Results of our MR study suggest that genetically determined Lp(a) levels are associated with parental life span and age at the end of the health span. We also provide evidence that genetically determined, as well as absolute Lp(a) levels, are associated with the long-term risk of all-cause and cardiovascular mortality in 18 720 participants of the EPIC-Norfolk prospective population study followed up for a mean of 20 years, in which the mortality risk for those with Lp(a) levels equal to or above the 95th percentile were equivalent to being 1.5 years older in chronologic age. Altogether, our results suggest that variants at *LPA*, through an increase in absolute Lp(a) levels, may be important determinants of human longevity.

Because the association between Lp(a) and longevity phenotypes is mostly related to its association with CVD mortality, one can speculate that Lp(a) may be a cause of premature mortality rather than the absence of Lp(a) being a cause of extreme longevity. Many pathobiological mechanisms have been proposed to explain the detrimental association of Lp(a) and health outcomes. First, Lp(a) is an important carrier of oxidized phospholipids in the bloodstream.^24^ Oxidized phospholipids are proinflammatory; they promote macrophage chemotaxis and oxidized phospholipid uptake within the arterial wall, where they also promote tissue necrosis.^25,26^ In addition, oxidized phospholipids have procalcifying properties. *LPA* is the top genetic loci for aortic stenosis, and studies have shown that Lp(a) was linked with aortic valve microcalcification in patients with and without aortic stenosis.^3,27^

In a 2017 genetic association study that sought to identify variants related to parental life span, Joshi et al^12^ identified 4 loci, including the *LPA* locus, to be associated with parental life span at the genome-wide significance level. In a follow-up study of more than 1 million parental life spans, Timmers et al^11^ confirmed the association between variants in *LPA* and parental life span. Interesting results were also recently reported by Zenin et al,^20^ who have suggested that variants in *LPA* may be associated with disease-free survival (also known as health span) in the UK Biobank, thereby suggesting that lower Lp(a) levels might not only be associated with longer life span but also with healthy living into old age. These studies, however, did not investigate the potential association of genetically elevated Lp(a) levels and parental life span or health span using robust genetic analyses, such as MR. By reporting a significant association of high Lp(a) levels with shorter parental life span and lower age at the end of the health span using MR in the 2 aforementioned studies, our study strengthens the possibility that Lp(a) is a potential causal determinant of human longevity.

Results of our study also provide support for the use of parental life span for the study of the genetic determinants of human longevity. The association between our trait of interest and parental life span reported herein using a 2-sample MR study design and subsequent validation in a long-term, prospective study that included 18 720 apparently healthy individuals with 5686 incident mortality cases also support the use of MR as a tool or surrogate to study the genetic makeup of human longevity. Mendelian randomization studies could be useful to determine whether suspected biological determinants of longevity have a potentially causal role in the genesis of this complex trait.

In 2009, the Emerging Risk Factor Collaboration reported a positive association between high Lp(a) levels and cardiovascular, but not all-cause, mortality in a meta-analysis of 24 long-term, prospective studies.^5^ More recently, investigators of 2 Danish prospective population studies (Copenhagen City Heart Study and Copenhagen General Population Study) also suggested a possible association between high levels of Lp(a) and all-cause and cardiovascular mortality in the general population.^8^ In these Danish studies, compared with participants in the bottom 50th percentile of the Lp(a) level distribution (all-cause mortality event rate of 14.2% and cardiovascular mortality event rate of 3.6%), participants with Lp(a) levels above the 95th percentile had an HR for all-cause mortality of 1.20 (95% CI, 1.10-1.30; event rate, 16.5%) and an HR for cardiovascular mortality of 1.50 (95% CI, 1.28-1.76; event rate, 5.0%). In our study using comparable subgroups, we found that the HRs for all-cause and cardiovascular mortality were consistent with the Danish studies. In our study, however, the absolute risk of all-cause and cardiovascular mortality in participants with Lp(a) levels above the 95th percentile was 8.6% higher for all-cause mortality and 8.7% higher for cardiovascular mortality than the group with low Lp(a) levels. The absolute risk associated with high Lp(a) levels reported herein is considerably higher than what was observed in the Copenhagen City Heart Study and Copenhagen General Population Study (2.5% for all-cause mortality and 1.4% for cardiovascular mortality). However, in contrast with the Copenhagen City Heart Study and Copenhagen General Population Study reports of a null association between the Lp(a)-raising variant rs10455872 and all-cause and cardiovascular mortality, we found a strong dose-response association between the number of rs10455872-G alleles and all-cause and cardiovascular mortality, thereby suggesting that absolute Lp(a) levels are associated with all-cause and cardiovascular mortality.

### Limitations

Limitations of our study include the use of individuals of European ancestry only. Confirmation of our findings that the high Lp(a) levels may influence mortality risk in other ethnic groups from different regions of the world will be needed to optimally plan randomized clinical trials of Lp(a) inhibition. We also only included patients from a primary prevention setting in EPIC-Norfolk. This study sample is not optimal to inform a randomized clinical trial design, which will likely be conducted in secondary prevention settings.

## Conclusions

Only a long-term clinical trial of Lp(a)-level lowering with investigative therapies will inform on the clinical benefits of change in risk or health trajectories of individuals with high Lp(a) levels. Under the assumption of a potential causal association between elevated Lp(a) levels and human longevity, our results provide support for the early identification and long-term treatment of individuals with elevated Lp(a) levels to promote life span as well as healthy living into old age.

## Notes

Supplement. **eMethods 1.** Parental Lifespan in the UK Biobank **eMethods 2.** Genotyping and Selection of Genetic Instruments **eTable 1.** Baseline Clinical Characteristics of the EPIC-Norfolk Study Population and the Study Population by Lipoprotein(a) Levels Percentiles **eTable 2.** Association Between Single Nucleotide Polymorphisms Included in the Weighted Genetic Risk Score Based on the Burgess et al Study and Lipoprotein(a) Levels and Longevity Phenotypes **eTable 3.** Association Between Single Nucleotide Polymorphisms Included in the Weighted Genetic Risk Score Based on the Mack et al Study and Unadjusted Lipoprotein(a) Levels and Longevity Phenotypes **eTable 4.** Association Between Single Nucleotide Polymorphisms Included in the Weighted Genetic Risk Score Based on the Mack et al Study and Lipoprotein(a) Levels Adjusted for Apolipoprotein(a) Isoform Size and Longevity Phenotypes **eReferences.** **eFigure 1.** Study Design **eFigure 2.** Distribution of Lipoprotein(a) Levels in the EPIC-Norfolk Study **eFigure 3.** Flowchart of the Parental Lifespan Outcome Definition in UK Biobank Analyses **eFigure 4.** Association Between Genetically Elevated Lipoprotein(a) Levels and Parental Lifespan in the UK Biobank **eFigure 5.** Mendelian Randomization Analysis of Genetically Elevated Lipoprotein(a) Levels and Longevity Phenotypes **eFigure 6.** Association Between Each Lipoprotein(a)-Raising Variant (Obtained From the Study of Burgess et al) and Longevity Phenotypes **eFigure 7.** Association Between Each Lipoprotein(a)-Raising Variant (Obtained From the Study of Mack et al) Without Adjusting for Apolipoprotein(a) Isoform Size and Longevity Phenotypes **eFigure 8.** Association Between Each Lipoprotein(a)-Raising Variant (Obtained From the Study of Mack et al) After Adjusting for Apolipoprotein(a) Isoform Size and Longevity Phenotypes. **eFigure 9.** Health Hazards Associated With High Lipoprotein(a) Levels in the EPIC-Norfolk Study by Baseline Age Categories **eFigure 10.** Impact of Lipoprotein(a) Levels on Noncardiovascular Disease Mortality in the EPIC-Norfolk **eFigure 11.** Event Rates and Hazard Ratios for All-Cause (A) and Cardiovascular Mortality (B) in Participants of the EPIC-Norfolk Study by Number of Lipoprotein(a)-Raising Alleles
