# Supplementary material for: Association of Long-term Exposure to Elevated Lipoprotein(a) Levels With Parental Life Span, Chronic Disease–Free Survival, and Mortality Risk: A Mendelian Randomization Analysis
Source: JAMA Netw Open. 2020 Feb 28;3(2):e200129. doi: 10.1001/jamanetworkopen.2020.0129 (PMC7049087; doi:10.1001/jamanetworkopen.2020.0129)
Supplement: Supplement. — eMethods 1. Parental Lifespan in the UK Biobank eMethods 2. Genotyping and Selection of Genetic Instruments eTable 1. Baseline Clinical Characteristics of the EPIC-Norfolk Study Population and the Study Population by Lipoprotein(a) Levels Percentiles eTable 2. Association Between Single Nucleotide Polymorphisms Included in the Weighted Genetic Risk Score Based on the Burgess et al Study and Lipoprotein(a) Levels and Longevity Phenotypes eTable 3. Association Between Single Nucleotide Polymorphisms Included in the Weighted Genetic Risk Score Based on the Mack et al Study and Unadjusted Lipoprotein(a) Levels and Longevity Phenotypes eTable 4. Association Between Single Nucleotide Polymorphisms Included in the Weighted Genetic Risk Score Based on the Mack et al Study and Lipoprotein(a) Levels Adjusted for Apolipoprotein(a) Isoform Size and Longevity Phenotypes eReferences. eFigure 1. Study Design eFigure 2. Distribution of Lipoprotein(a) Levels in the EPIC-Norfolk Study eFigure 3. Flowchart of the Parental Lifespan Outcome Definition in UK Biobank Analyses eFigure 4. Association Between Genetically Elevated Lipoprotein(a) Levels and Parental Lifespan in the UK Biobank eFigure 5. Mendelian Randomization Analysis of Genetically Elevated Lipoprotein(a) Levels and Longevity Phenotypes eFigure 6. Association Between Each Lipoprotein(a)-Raising Variant (Obtained From the Study of Burgess et al) and Longevity Phenotypes eFigure 7. Association Between Each Lipoprotein(a)-Raising Variant (Obtained From the Study of Mack et al) Without Adjusting for Apolipoprotein(a) Isoform Size and Longevity Phenotypes eFigure 8. Association Between Each Lipoprotein(a)-Raising Variant (Obtained From the Study of Mack et al) After Adjusting for Apolipoprotein(a) Isoform Size and Longevity Phenotypes. eFigure 9. Health Hazards Associated With High Lipoprotein(a) Levels in the EPIC-Norfolk Study by Baseline Age Categories eFigure 10. Impact of Lipoprotein(a) Levels on Noncardiovascular Disease Mo [file jamanetwopen-3-e200129-s001.pdf]

Supplementary Online Content

Arsenault BJ, Pelletier W, Kaiser Y, et al. Association of long-term exposure to elevated lipoprotein(a) levels with parental life span, chronic disease–free survival, and mortality risk: a mendelian randomization analysis. *JAMA Netw Open*. 2020;3(2):e200129. doi:10.1001/jamanetworkopen.2020.0129

eMethods 1. Parental Lifespan in the UK Biobank
eMethods 2. Genotyping and Selection of Genetic Instruments
eTable 1. Baseline Clinical Characteristics of the EPIC-Norfolk Study Population and the Study Population by Lipoprotein(a) Levels Percentiles
eTable 2. Association Between Single Nucleotide Polymorphisms Included in the Weighted Genetic Risk Score Based on the Burgess et al Study and Lipoprotein(a) Levels and Longevity Phenotypes
eTable 3. Association Between Single Nucleotide Polymorphisms Included in the Weighted Genetic Risk Score Based on the Mack et al Study and Unadjusted Lipoprotein(a) Levels and Longevity Phenotypes
eTable 4. Association Between Single Nucleotide Polymorphisms Included in the Weighted Genetic Risk Score Based on the Mack et al Study and Lipoprotein(a) Levels Adjusted for Apolipoprotein(a) Isoform Size and Longevity Phenotypes
eReferences.
eFigure 1. Study Design
eFigure 2. Distribution of Lipoprotein(a) Levels in the EPIC-Norfolk Study
eFigure 3. Flowchart of the Parental Lifespan Outcome Definition in UK Biobank Analyses
eFigure 4. Association Between Genetically Elevated Lipoprotein(a) Levels and Parental Lifespan in the UK Biobank
eFigure 5. Mendelian Randomization Analysis of Genetically Elevated Lipoprotein(a) Levels and Longevity Phenotypes
eFigure 6. Association Between Each Lipoprotein(a)-Raising Variant (Obtained From the Study of Burgess et al) and Longevity Phenotypes
eFigure 7. Association Between Each Lipoprotein(a)-Raising Variant (Obtained From the Study of Mack et al) Without Adjusting for Apolipoprotein(a) Isoform Size and Longevity Phenotypes
eFigure 8. Association Between Each Lipoprotein(a)-Raising Variant (Obtained From the Study of Mack et al) After Adjusting for Apolipoprotein(a) Isoform Size and Longevity Phenotypes.
eFigure 9. Health Hazards Associated With High Lipoprotein(a) Levels in the EPIC-Norfolk Study by Baseline Age Categories
eFigure 10. Impact of Lipoprotein(a) Levels on Noncardiovascular Disease Mortality in the EPIC-Norfolk
eFigure 11. Event Rates and Hazard Ratios for All-Cause (A) and Cardiovascular Mortality (B) in Participants of the EPIC-Norfolk Study by Number of Lipoprotein(a)-Raising Alleles

This supplementary material has been provided by the authors to give readers additional information about their work.

© 2020 Arsenault BJ et al. *JAMA Network Open.*

eMethods 1: Parental lifespan in the UK Biobank

We used the definition of Pilling et al. (1) to define high parental lifespan in participants of the UK Biobank. Only participants between 55 and 69 years were included. Participants who were adopted, had missing information on age of parents' death or who had parents who died at a young age (<46 for the father and <57 for the mother) were excluded from these analyses. Parents were separated into three categories: long-lived (father still alive and older than 90 or father's age of death ≥90 and mother still alive and older than 93 or mother's age of death ≥93), medium lived (age of death ≥66 and <89 for the father and ≥73 and <92 for the mother) and short lived (age of death ≥46 and <65 for the father and ≥57 and <72 for the mother). We defined high parental lifespan as at least one long-lived parent (i.e. long/long or long/medium). Analyses were also performed for high paternal lifespan (with maternal lifespan being long or medium) and for high maternal lifespan (with paternal lifespan being long or medium). A second, more stringent, outcome was defined as at least one parent with exceptional longevity (top 1% survival, i.e. age of death ≥95 for the father or ≥98 for the mother with the other parent either long- or medium-lived). The control group included participants with parents considered as short- or medium-lived (i.e. short/short, short/medium or medium/medium). Participants discordant for mothers' and fathers' age of death (one long-lived parent and one short-lived parent) were also excluded from the present analyses. eFigure 2 presents the flowchart of the UK Biobank analysis.

eMethods 2: Genotyping and selection of genetic instruments

Samples were genotyped with the Affymetrix UK BiLEVE Axiom array or the Affymetrix UK Biobank Axiom Array. On the UK Biobank, phasing and imputation were performed centrally using the Haplotype Reference Consortium (HRC) reference panel (2). Samples with call rate <95%, outlier heterozygosity rate, gender mismatch, non-white British ancestry, related samples (second degree or closer), samples with excess third-degree relatives (>10), or not used for relatedness calculation were excluded. Burgess et al. (3) recently used a genetic risk score (GRS) of 43 single nucleotide polymorphisms (SNP) that explained approximately 60% of the variance in Lp(a) levels in four large cohorts (R^2 measure of linkage disequilibrium <0.4). The marginal effect of these SNPs on Lp(a) levels was obtained. To derive an estimation of genetically-determined Lp(a) levels, we included 26 SNPs from the report of Burgess et al.(3) that had a minor allele frequency equal or above 0.005. We also only included independent SNPs (R^2<0.2). We built weighted genetic risk score using these 26 SNPs weighted by the effect of each SNP on Lp(a) levels. The analyses presented in the main paper were obtained from this wGRS. We engineered two additional wGRS based on the genome-wide association study of Mack et al. (4) who identified SNPs associated with Lp(a) and Lp(a) adjusted for apolipoprotein(a) isoform size from five European studies. The genetic instruments are presented in eTable 2, 3 and 4. In EPIC-Norfolk, we used two SNPs that had the strongest impact on Lp(a) levels (rs10455872 and rs3798220) in the study of Clarke et al (5).

© 2020 Arsenault BJ et al. *JAMA Network Open.*

eTable 1. Baseline clinical characteristics of the EPIC-Norfolk study population and the study population by Lipoprotein(a) levels percentiles.

	Lp(a) percentile				
	<50	50-79	80-89	90-94	>=95
Lp(a) range (mg/dL)	0.01-11.41	11.42-35.03	35.05-53.32	53.34-69.70	69.73-174.96
Age, years	58.6 (±9.2)	59.9 (±9.1)	58.9 (±9.2)	59.7 (±9.1)	60.5 (±8.9)
Male, % (N)	45.8 (5078)	44.1 (3138)	46.3 (1004)	41.0 (553)	39.7 (564)
Active smokers, % (N)	11.6 (1074)	11.3 (627)	9.8 (181)	11.4 (106)	12.1 (112)
Body mass index, kg/m^2	26.3 (±3.9)	26.3 (±3.7)	26.1 (±3.7)	26.3 (±3.9)	26.1 (±3.6)
Systolic blood pressure, mmHg	134.6 (±18.1)	136.1 (±18.6)	134.5 (18.4)	134.9 (±18.3)	134.8 (±17.7)
Diastolic blood pressure, mmHg	82.1 (±11.2)	82.6 (±11.2)	82.1 (±11.3)	82.1 (±10.8)	81.7 (±10.9)
Diabetes mellitus, % (N)	2.3 (212)	2.0 (114)	1.8 (33)	2.2 (21)	2.8 (26)
Total cholesterol, mmol/l	6.0 (±1.1)	6.3 (±1.2)	6.2 (±1.1)	6.6 (±1.2)	6.8 (±1.1)
LDL cholesterol, mmol/l	3.8 (±1.0)	4.1 (±1.0)	4.0 (±1.0)	4.3 (±1.0)	4.5 (±1.0)
HDL cholesterol, mmol/l	1.4 (±0.4)	1.4 (±0.4)	1.4 (±0.5)	1.5 (±0.4)	1.5 (±0.4)
Triglycerides, median (IQR)	1.5 (1.1-2.3)	1.5 (1.1-2.2)	1.5 (1.1-2.1)	1.6 (1.1-2.3)	1.5 (1.1-2.1)
Creatinine, μmol/l	85.7 (±19.9)	88.0 (±24.8)	86.6 (±18.5)	87.8 (±18.0)	88.2 (±24.0)

Data are presented as mean (standard deviation) or median (interquartile range) for continuous variables and as % (N) for categorical variables. Lp(a) indicates lipoprotein(a), LDL indicates low-density lipoprotein, HDL indicates high-density lipoprotein and IQR indicates interquartile range.

© 2020 Arsenault BJ et al. *JAMA Network Open.*

eTable 2. Association between single nucleotide polymorphisms included in the weighted genetic risk score based on the Burgess et al. study and lipoprotein(a) levels and longevity phenotypes.

Chromosome: Position (GRCh37/hg19)	rsID	Effect Allele	Other Allele	Effect allele frequency	Lp(a) beta [SE]	Lp(a) pvalue	UKB lifespan beta [SE]	UKB lifespan pvalue	UKB + LifeGEN lifespan beta [SE]	UKB + LifeGEN lifespan pvalue	UKB Healthspan beta [SE]	UKB Healthspan pvalue
6:161010118	rs10455872	G	A	0,072	46,2 [0,4]	NA	-0,091 [0,022]	2,39E-05	-0,076 [0,007]	8,54E-25	-0,057 [0,009]	1,42E-10
6:160847571	rs117446263	A	G	0,022	19,6 [0,8]	NA	-0,018 [0,037]	6,28E-01	-0,05 [0,013]	1,30E-04	-0,009 [0,016]	5,81E-01
6:161177443	rs117534432	T	C	0,036	2,6 [0,7]	NA	-0,022 [0,032]	4,96E-01	0,001 [0,011]	9,34E-01	-0,022 [0,014]	1,05E-01
6:161189071	rs11753588	G	A	0,891	5,9 [0,4]	NA	-0,027 [0,018]	1,38E-01	-0,007 [0,006]	2,95E-01	0,007 [0,008]	3,43E-01
6:161251940	rs138491411	G	A	0,012	7,2 [1,2]	NA	-0,095 [0,057]	9,74E-02	-0,053 [0,021]	1,16E-02	-0,008 [0,024]	7,46E-01
6:161135746	rs139389770	G	T	0,011	5,5 [1,2]	NA	-0,075 [0,056]	1,83E-01	-0,014 [0,021]	4,85E-01	-0,044 [0,023]	5,82E-02
6:160909667	rs141834709	A	T	0,009	22 [1,3]	NA	-0,004 [0,066]	9,57E-01	-0,03 [0,025]	2,44E-01	-0,048 [0,029]	9,35E-02
6:160942926	rs142126734	A	G	0,049	4,9 [0,6]	NA	-0,027 [0,026]	3,03E-01	-0,004 [0,009]	6,65E-01	0,008 [0,011]	4,76E-01
6:160825930	rs143365644	A	T	0,965	3,8 [0,7]	NA	0,041 [0,034]	2,30E-01	0,012 [0,011]	3,11E-01	0 [0,014]	9,77E-01
6:160954800	rs143461353	T	C	0,008	8,3 [1,4]	NA	0,093 [0,061]	1,29E-01	-0,024 [0,024]	3,12E-01	-0,006 [0,027]	8,28E-01
6:160891897	rs182443492	A	C	0,009	36,3 [1,4]	NA	-0,146 [0,06]	1,58E-02	-0,108 [0,02]	5,16E-08	-0,109 [0,025]	8,97E-06
6:161257953	rs34371670	C	T	0,984	11 [1]	NA	0,02 [0,045]	6,51E-01	-0,038 [0,016]	1,48E-02	0,023 [0,019]	2,21E-01
6:160961137	rs3798220	C	T	0,014	51,2 [1]	NA	-0,148 [0,045]	9,72E-04	-0,092 [0,015]	4,18E-10	-0,101 [0,018]	3,59E-08
6:161022107	rs41259144	C	T	0,989	12,8 [1,2]	NA	0,058 [0,056]	3,02E-01	-0,019 [0,021]	3,64E-01	-0,013 [0,024]	5,97E-01
6:160953137	rs41266379	C	T	0,02	8,5 [0,9]	NA	0,025 [0,044]	5,75E-01	0,001 [0,015]	9,22E-01	0,04 [0,019]	3,66E-02
6:160953642	rs41267809	A	G	0,978	12,4 [0,9]	NA	-0,075 [0,039]	5,64E-02	-0,025 [0,014]	7,05E-02	-0,022 [0,017]	2,04E-01

© 2020 Arsenault BJ et al. *JAMA Network Open.*

6:161070653	rs41269876	C	A	0,972	10,5 [0,7]	NA	0,008 [0,036]	8,13E-01	-0,014 [0,012]	2,66E-01	-0,001 [0,015]	9,56E-01
6:161159366	rs4252152	G	T	0,014	8,5 [1,1]	NA	-0,006 [0,062]	9,19E-01	-0,037 [0,02]	6,16E-02	-0,015 [0,026]	5,62E-01
6:161162406	rs4252170	C	T	0,082	4,2 [0,5]	NA	0,002 [0,021]	9,42E-01	-0,004 [0,007]	6,16E-01	-0,017 [0,009]	5,62E-02
6:161285760	rs4709474	G	A	0,49	4,9 [0,2]	NA	-0,003 [0,011]	7,77E-01	-0,011 [0,004]	5,59E-03	-0,017 [0,005]	5,98E-04
6:160890350	rs6916433	A	T	0,86	4,1 [0,4]	NA	0,028 [0,017]	1,10E-01	-0,003 [0,006]	5,91E-01	0 [0,007]	9,73E-01
6:161017363	rs73596816	A	G	0,034	22,1 [0,7]	NA	-0,057 [0,032]	8,18E-02	-0,023 [0,011]	4,30E-02	-0,013 [0,014]	3,50E-01
6:161088956	rs75274517	A	G	0,01	1,4 [1,2]	NA	0,055 [0,052]	2,86E-01	-0,027 [0,019]	1,68E-01	0,015 [0,022]	4,94E-01
6:161087652	rs77337569	T	G	0,987	2,2 [1,1]	NA	0,007 [0,056]	8,95E-01	-0,033 [0,02]	9,91E-02	-0,029 [0,024]	2,19E-01
6:161078894	rs79246098	C	T	0,01	0,9 [1,3]	NA	0,136 [0,062]	2,94E-02	0,05 [0,024]	3,82E-02	0,03 [0,028]	2,73E-01
6:161012805	rs9456551	T	C	0,65	5,9 [0,3]	NA	-0,026 [0,012]	2,95E-02	-0,016 [0,004]	1,27E-04	-0,004 [0,005]	4,00E-01

© 2020 Arsenault BJ et al. *JAMA Network Open.*

eTable 3. Association between single nucleotide polymorphisms included in the weighted genetic risk score based on the Mack et al. study and unadjusted lipoprotein(a) levels and longevity phenotypes.

Chromosome: Position (GRCh37/hg19)	rsID	Effect Allele	Other Allele	Effect allele frequency	Lp(a) beta [SE]	Lp(a) pvalue	UKB lifespan beta [SE]	UKB lifespan pvalue	UKB + LifeGEN lifespan beta [SE]	UKB + LifeGEN lifespan pvalue	UKB Healthspan beta [SE]	UKB Healthspan pvalue
6:160985526	rs118039278	A	G	0,074	32,4 [0,7]	1,36e-396	-0,092 [0,022]	2,13E-05	-0,076 [0,007]	9,18E-25	-0,057 [0,009]	2,42E-10
6:160799246	rs12204009	C	T	0,009	32,9 [2,3]	2,80E-51	-0,126 [0,087]	1,46E-01			-0,141 [0,035]	5,82E-05
6:161125631	rs12529361	C	T	0,217	5 [0,4]	5,57E-69	-0,002 [0,014]	8,66E-01	-0,004 [0,005]	4,49E-01	-0,013 [0,006]	2,73E-02
6:160948136	rs12664092	C	A	0,028	5,4 [0,8]	1,92E-07	0,005 [0,038]	8,91E-01	-0,006 [0,013]	6,72E-01	0,033 [0,016]	4,12E-02
6:161221380	rs139699952	C	G	0,046	6,8 [1,2]	4,70E-11	0,008 [0,031]	7,89E-01	-0,033 [0,011]	2,32E-03	-0,001 [0,013]	9,13E-01
6:160838646	rs141463285	A	T	0,007	24,1 [2,6]	6,28E-18	0,02 [0,081]	8,01E-01			-0,067 [0,035]	5,18E-02
6:160942926	rs142126734	A	G	0,042	6,6 [0,7]	8,52E-48	-0,027 [0,026]	3,03E-01	-0,004 [0,009]	6,65E-01	0,008 [0,011]	4,76E-01
6:160631170	rs143520616	T	G	0,032	2,4 [1,2]	5,01E-08	0,039 [0,037]	3,01E-01	0,024 [0,013]	6,35E-02	-0,02 [0,016]	2,24E-01
6:160867272	rs143665477	C	T	0,008	39,7 [2,6]	2,63E-41	-0,067 [0,054]	2,18E-01	-0,076 [0,019]	8,59E-05	-0,012 [0,023]	5,88E-01
6:161177756	rs184278183	T	C	0,003	26,3 [1,7]	9,36E-34	-0,152 [0,066]	2,04E-02	-0,127 [0,023]	2,67E-08	-0,102 [0,027]	1,16E-04
6:161111700	rs186696265	T	C	0,011	64,7 [1,3]	4,15E-231	-0,172 [0,051]	6,68E-04	-0,136 [0,017]	2,15E-16	-0,094 [0,02]	3,78E-06
6:161508438	rs2140650	G	A	0,217	0,9 [0,3]	1,10E-03	-0,002 [0,013]	8,89E-01	-0,003 [0,004]	4,90E-01	0,003 [0,006]	6,00E-01
6:161022107	rs41259144	C	T	0,987	14,3 [1,9]	4,45E-24	0,058 [0,056]	3,02E-01	-0,019 [0,021]	3,64E-01	-0,013 [0,024]	5,97E-01
6:161087863	rs41269133	T	C	0,885	5,7 [0,5]	5,42E-55	-0,007 [0,021]	7,30E-01	-0,027 [0,007]	1,34E-04	-0,019 [0,009]	2,83E-02
6:161006077	rs41272114	C	T	0,972	8,6 [0,8]	4,74E-86	-0,042 [0,03]	1,58E-01	-0,027 [0,01]	9,70E-03	-0,014 [0,013]	2,84E-01
6:160767905	rs520829	T	G	0,519	3,6 [0,3]	2,65E-29	-0,013 [0,011]	2,61E-01	-0,016 [0,004]	4,15E-05	-0,018 [0,005]	1,54E-04
6:160505199	rs614754	C	G	0,016	16,1 [2]	1,43E-15	0,034 [0,048]	4,70E-01	-0,052 [0,017]	2,37E-03	-0,031 [0,021]	1,34E-01
6:160569068	rs62440901	T	C	0,148	2,1 [0,4]	3,11E-14	-0,002 [0,016]	8,86E-01	-0,001 [0,005]	8,47E-01	-0,016 [0,007]	1,97E-02
6:160987060	rs62441903	A	G	0,983	14,4 [1,7]	4,86E-33	-0,092 [0,055]	9,52E-02	-0,02 [0,02]	3,10E-01	-0,035 [0,024]	1,44E-01
6:160986915	rs6938647	C	A	0,781	5,5 [0,4]	7,48E-119	-0,016 [0,014]	2,56E-01	-0,008 [0,005]	9,36E-02	-0,025 [0,006]	2,47E-05
6:161017363	rs73596816	A	G	0,021	11,2 [1,7]	1,53E-18	-0,057 [0,032]	8,18E-02	-0,023 [0,011]	4,30E-02	-0,013 [0,014]	3,50E-01
6:160985501	rs76000021	T	C	0,984	7,5 [1,3]	4,70E-13	0,037 [0,05]	4,59E-01	-0,03 [0,017]	6,85E-02	0,019 [0,021]	3,58E-01
6:161078894	rs79246098	C	T	0,011	0,2 [1,6]	7,53E-05	0,136 [0,062]	2,94E-02	0,05 [0,024]	3,82E-02	0,03 [0,028]	2,73E-01
6:160353291	rs9457778	C	T	0,781	0,1 [0,3]	8,75E-04	0,007 [0,015]	6,54E-01	0,01 [0,005]	4,83E-02	0 [0,006]	9,64E-01

© 2020 Arsenault BJ et al. *JAMA Network Open.*

eTable 4. Association between single nucleotide polymorphisms included in the weighted genetic risk score based on the Mack et al. study and lipoprotein(a) levels adjusted for apolipoprotein(a) isoform size and longevity phenotypes.

Chromosome: Position (GRCh37/hg19)	rsID	Effect Allele	Other Allele	Effect allele frequency	Lp(a) beta [SE]	Lp(a) pvalue	UKB lifespan beta [SE]	UKB lifespan pvalue	UKB + LifeGEN lifespan beta [SE]	UKB + LifeGEN lifespan pvalue	UKB Healthspan beta [SE]	UKB Healthspan pvalue
6:160955713	rs112842440	T	G	0,016	6,9 [1]	1,06E-23	0 [0,04]	9,99E-01	0,022 [0,014]	1,22E-01	-0,004 [0,017]	8,26E-01
6:161068235	rs117026595	A	T	0,976	13,8 [1,5]	2,61E-28	0,029 [0,058]	6,10E-01	0,004 [0,02]	8,45E-01	0,016 [0,024]	5,05E-01
6:160903911	rs12207325	G	A	0,995	14,8 [1,5]	2,50E-37	-0,18 [0,054]	9,27E-04	-0,037 [0,02]	7,05E-02	0,035 [0,024]	1,46E-01
6:160948136	rs12664092	C	A	0,028	7,2 [0,7]	1,72E-23	0,005 [0,038]	8,91E-01	-0,006 [0,013]	6,72E-01	0,033 [0,016]	4,12E-02
6:161013013	rs140570886	C	T	0,011	43,4 [1]	1,72E-210	-0,189 [0,05]	1,64E-04	-0,127 [0,016]	4,80E-15	-0,116 [0,02]	9,23E-09
6:160838646	rs141463285	A	T	0,007	18 [2,2]	3,27E-15	0,02 [0,081]	8,01E-01			-0,067 [0,035]	5,18E-02
6:160942926	rs142126734	A	G	0,042	7,8 [0,6]	2,70E-57	-0,027 [0,026]	3,03E-01	-0,004 [0,009]	6,65E-01	0,008 [0,011]	4,76E-01
6:161004351	rs145470851	G	A	0,991	16 [1,4]	8,83E-34	-0,076 [0,067]	2,57E-01	-0,022 [0,023]	3,32E-01	0,015 [0,029]	5,96E-01
6:160625299	rs147010904	T	C	0,004	18,4 [1,8]	9,25E-20	-0,127 [0,063]	4,34E-02	-0,093 [0,022]	2,10E-05	-0,063 [0,026]	1,50E-02
6:160998148	rs3798221	G	T	0,788	1,3 [0,4]	0,0106	-0,019 [0,014]	1,79E-01	-0,019 [0,005]	6,23E-05	-0,01 [0,006]	1,03E-01
6:160952816	rs41267807	T	C	0,983	5,3 [1]	3,25E-08	0,01 [0,042]	8,12E-01	-0,027 [0,015]	5,90E-02	0,007 [0,018]	7,00E-01
6:160953642	rs41267809	A	G	0,979	6,8 [1,1]	2,95E-11	-0,075 [0,039]	5,64E-02	-0,025 [0,014]	7,05E-02	-0,022 [0,017]	2,04E-01
6:161006077	rs41272114	C	T	0,972	5,7 [0,7]	2,97E-24	-0,042 [0,03]	1,58E-01	-0,027 [0,01]	9,70E-03	-0,014 [0,013]	2,84E-01
6:161005610	rs55730499	T	C	0,07	16,8 [0,6]	3,97E-163	-0,094 [0,021]	1,09E-05	-0,076 [0,007]	1,09E-24	-0,057 [0,009]	1,81E-10
6:160569068	rs62440901	T	C	0,148	0,9 [0,3]	0,00465	-0,002 [0,016]	8,86E-01	-0,001 [0,005]	8,47E-01	-0,016 [0,007]	1,97E-02
6:160986915	rs6938647	C	A	0,781	5,5 [0,3]	2,36E-87	-0,016 [0,014]	2,56E-01	-0,008 [0,005]	9,36E-02	-0,025 [0,006]	2,47E-05

© 2020 Arsenault BJ et al. *JAMA Network Open.*

6:161018985	rs75234242	G	A	0,963	13,6 [0,7]	3,78E-97	-0,003 [0,034]	9,30E-01	-0,001 [0,012]	9,48E-01	0,011 [0,014]	4,58E-01
6:161030231	rs75692336	C	A	0,858	9,7 [0,4]	2,90E-216	-0,012 [0,017]	4,59E-01	-0,002 [0,006]	7,61E-01	0,011 [0,007]	1,23E-01
6:160865645	rs7769879	C	G	0,358	6,7 [0,2]	4,43E-168	-0,008 [0,012]	5,06E-01	-0,018 [0,004]	4,68E-06	-0,024 [0,005]	1,71E-06
6:161654115	rs9295143	G	C	0,047	3 [0,6]	8,23E-09	0,019 [0,026]	4,63E-01	-0,013 [0,009]	1,59E-01	-0,014 [0,011]	1,90E-01

© 2020 Arsenault BJ et al. *JAMA Network Open.*

eReferences

1. Pilling LC, Atkins J, Bowman K et al. Human longevity is influenced by many genetic variants: evidence from 75,000 UK Biobank participants. AGING-US 2016;8:547-563.
2. McCarthy S, Das S, Kretzschmar W et al. A reference panel of 64,976 haplotypes for genotype imputation. Nat Genet 2016;48:1279-83.
3. Burgess S, Ference BA, Staley JR et al. Association of LPA Variants With Risk of Coronary Disease and the Implications for Lipoprotein(a)-Lowering Therapies: A Mendelian Randomization Analysis. JAMA cardiology 2018;3:619.
4. Mack S, Coassin S, Rueedi R et al. A genome-wide association meta-analysis on lipoprotein (a) concentrations adjusted for apolipoprotein (a) isoforms [S]. Journal of Lipid Research 2017;58:1834-1844.
5. Clarke R, Peden JF, Hopewell JC et al. Genetic variants associated with Lp(a) lipoprotein level and coronary disease. N Engl J Med 2009;361:2518-28.

© 2020 Arsenault BJ et al. *JAMA Network Open.*

eFigure 1. Study design.

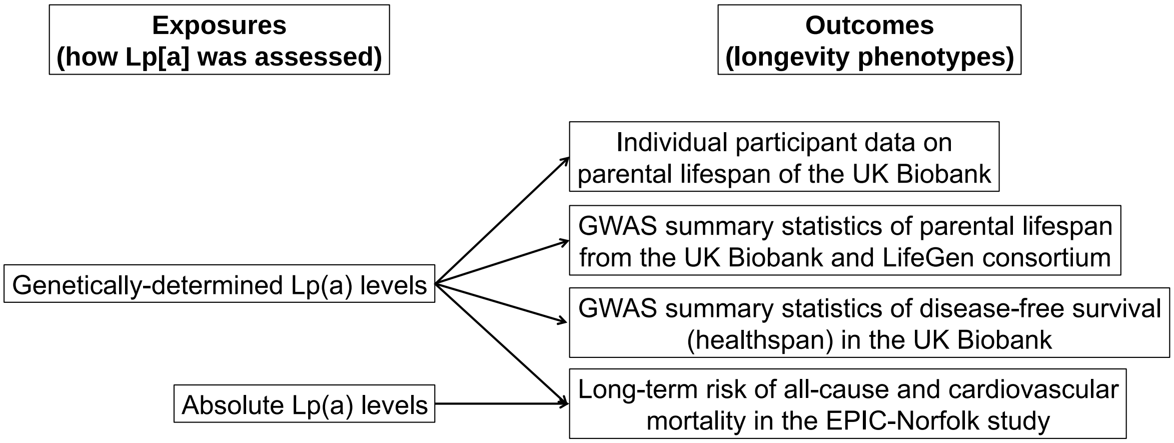

© 2020 Arsenault BJ et al. *JAMA Network Open.*

eFigure 2. Distribution of Lipoprotein(a) levels in the EPIC-Norfolk study.

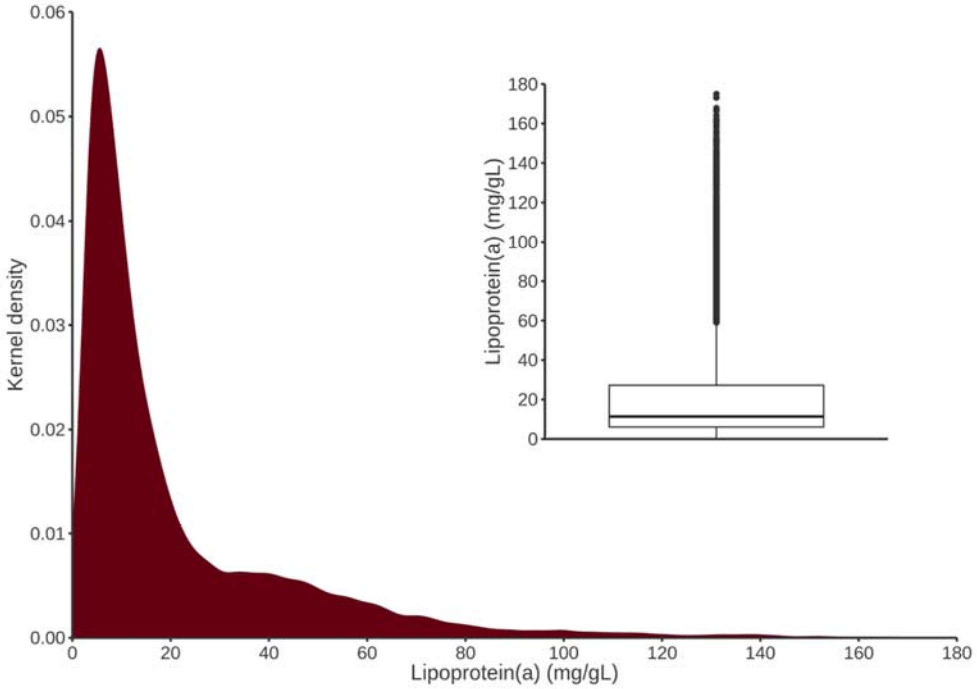

© 2020 Arsenault BJ et al. *JAMA Network Open.*

eFigure 3. Flowchart of the parental lifespan outcome definition in UK Biobank analyses.

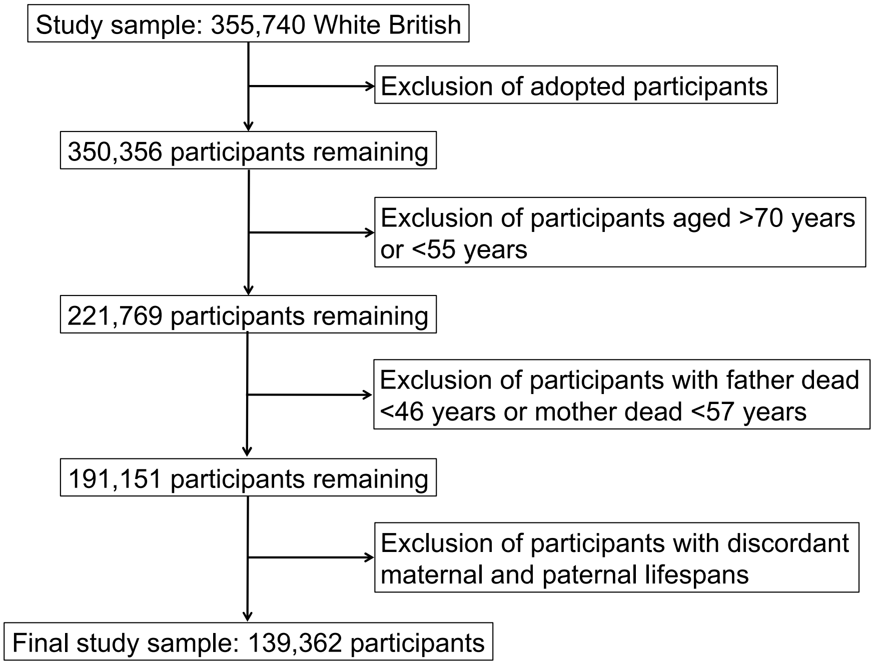

© 2020 Arsenault BJ et al. *JAMA Network Open.*

eFigure 4. Association between genetically elevated lipoprotein(a) levels and longevity phenotypes.

A)

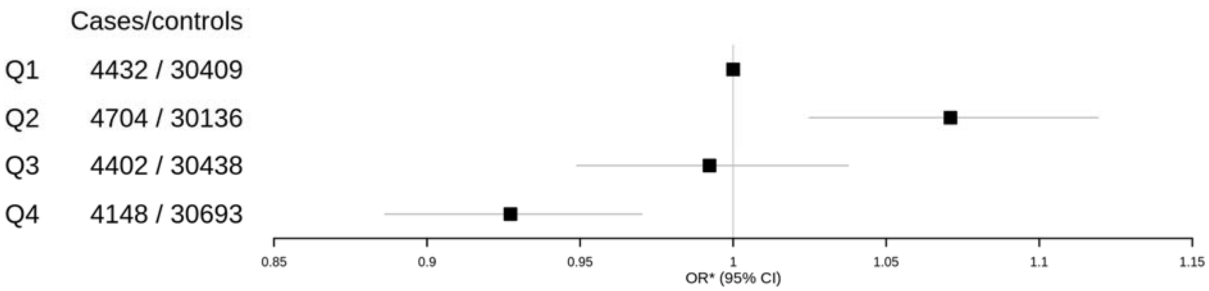

B)

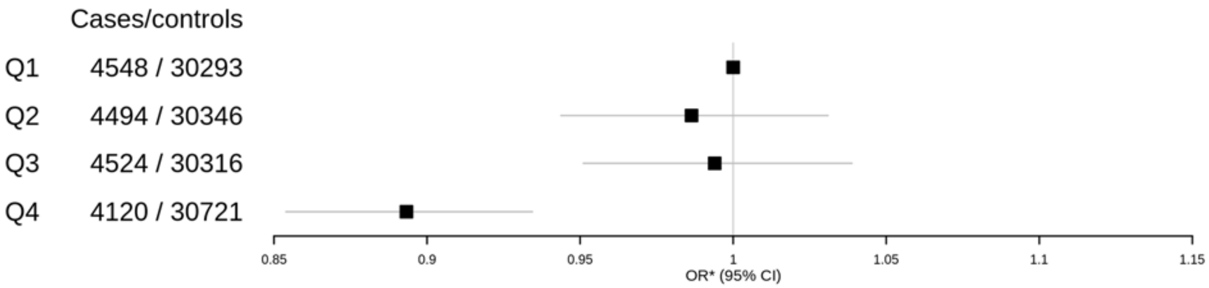

C)

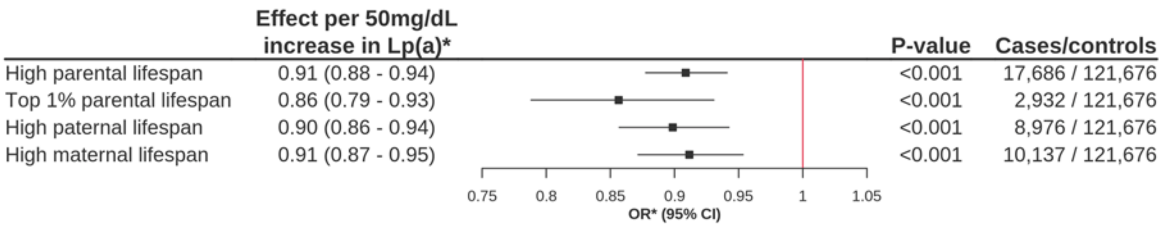

© 2020 Arsenault BJ et al. *JAMA Network Open.*

D)

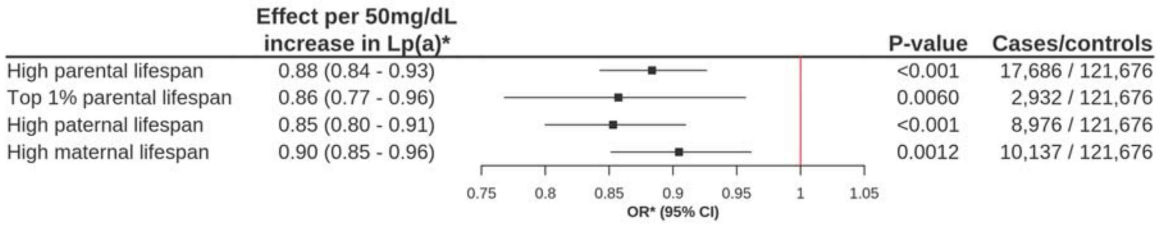

E)

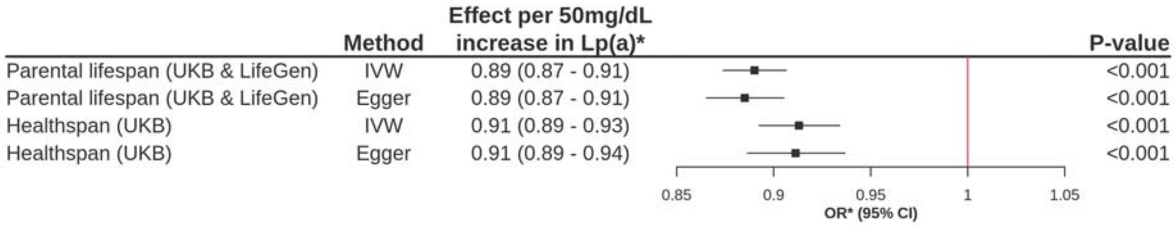

F)

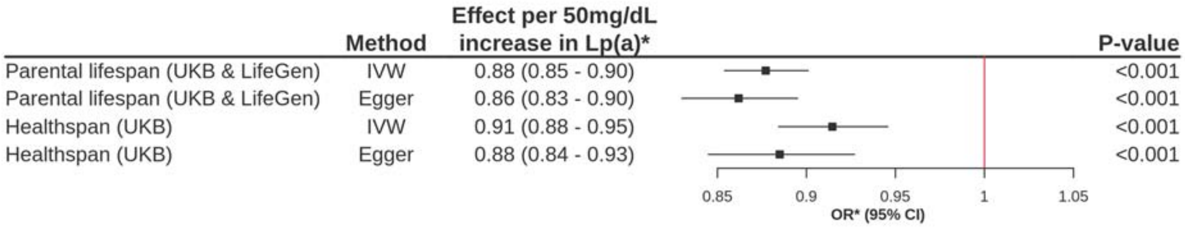

Odds ratios (OR) and 95% confidence interval (CI) are presented for high parental lifespan in participants of the UK Biobank separated into quartiles of the *LPA* weighted genetic risk score (wGRS) adjusted for age, sex and the 10 first ancestry-based principal components (A) and further adjusted for apolipoprotein(a) isoform size from Mack et al. (B). ORs and 95% CI for high parental lifespan, top 1% parental lifespan, high paternal lifespan and high maternal lifespan associated with a 50 mg/dL increase in the *LPA* wGRS in the UK Biobank from Mack et al. adjusted for age, sex and the 10 first ancestry-based principal components (C) and further adjusted for apolipoprotein(a) isoform size from Mack et al. (D) are also shown. Finally, the OR and 95% CI for parental lifespan (UK Biobank and LifeGen consortium) and the age at the end of healthspan. associated with a 50 mg/dL increase in the *LPA* wGRS from Mack et al. adjusted for age, sex and the 10 first ancestry-based principal components (E) and further adjusted for apolipoprotein(a) isoform size from Mack et al. (F) are also shown.

© 2020 Arsenault BJ et al. *JAMA Network Open.*

eFigure 5. Mendelian randomization analysis of genetically elevated lipoprotein(a) levels and longevity phenotypes.
A)

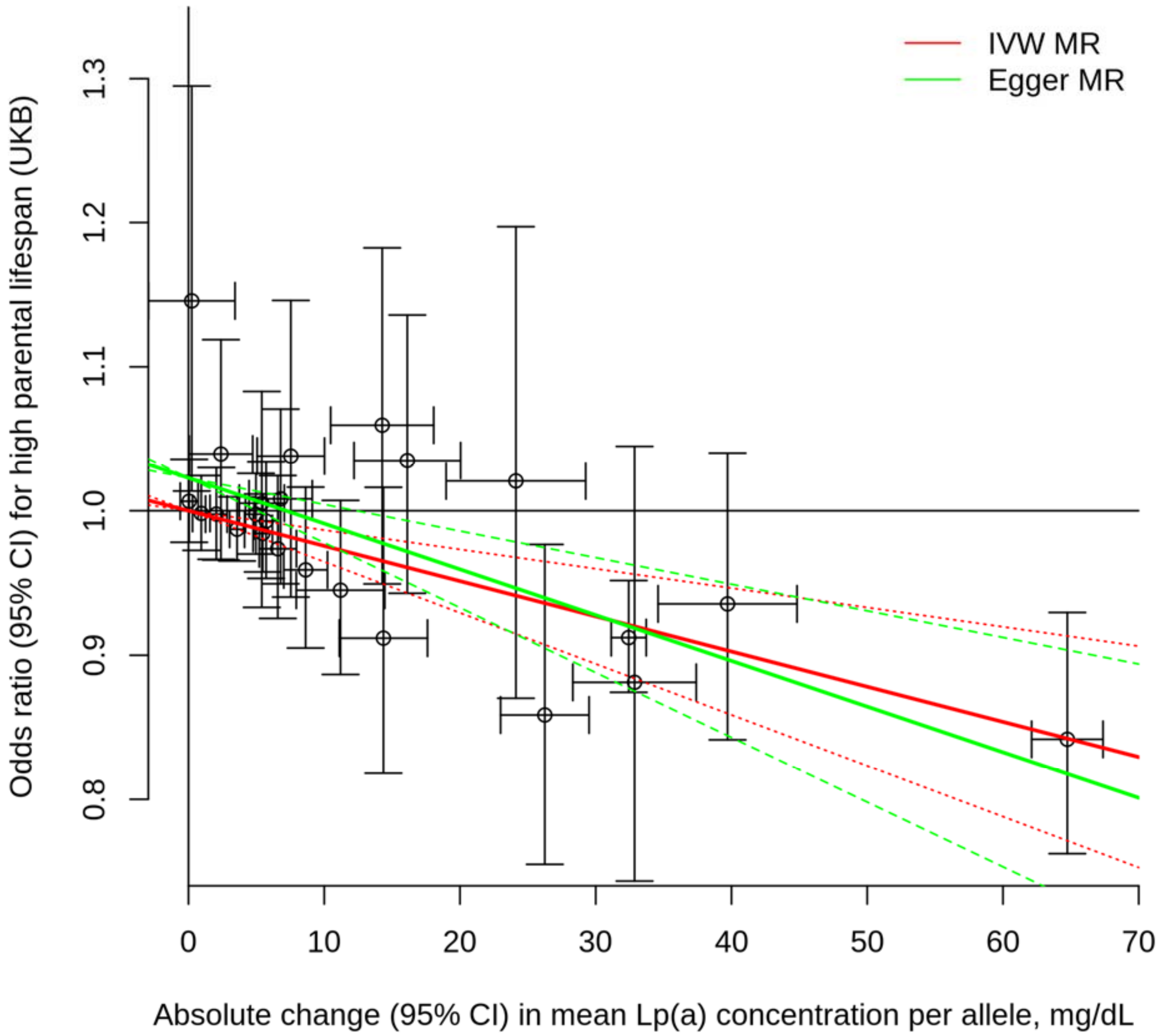

© 2020 Arsenault BJ et al. *JAMA Network Open.*

B)

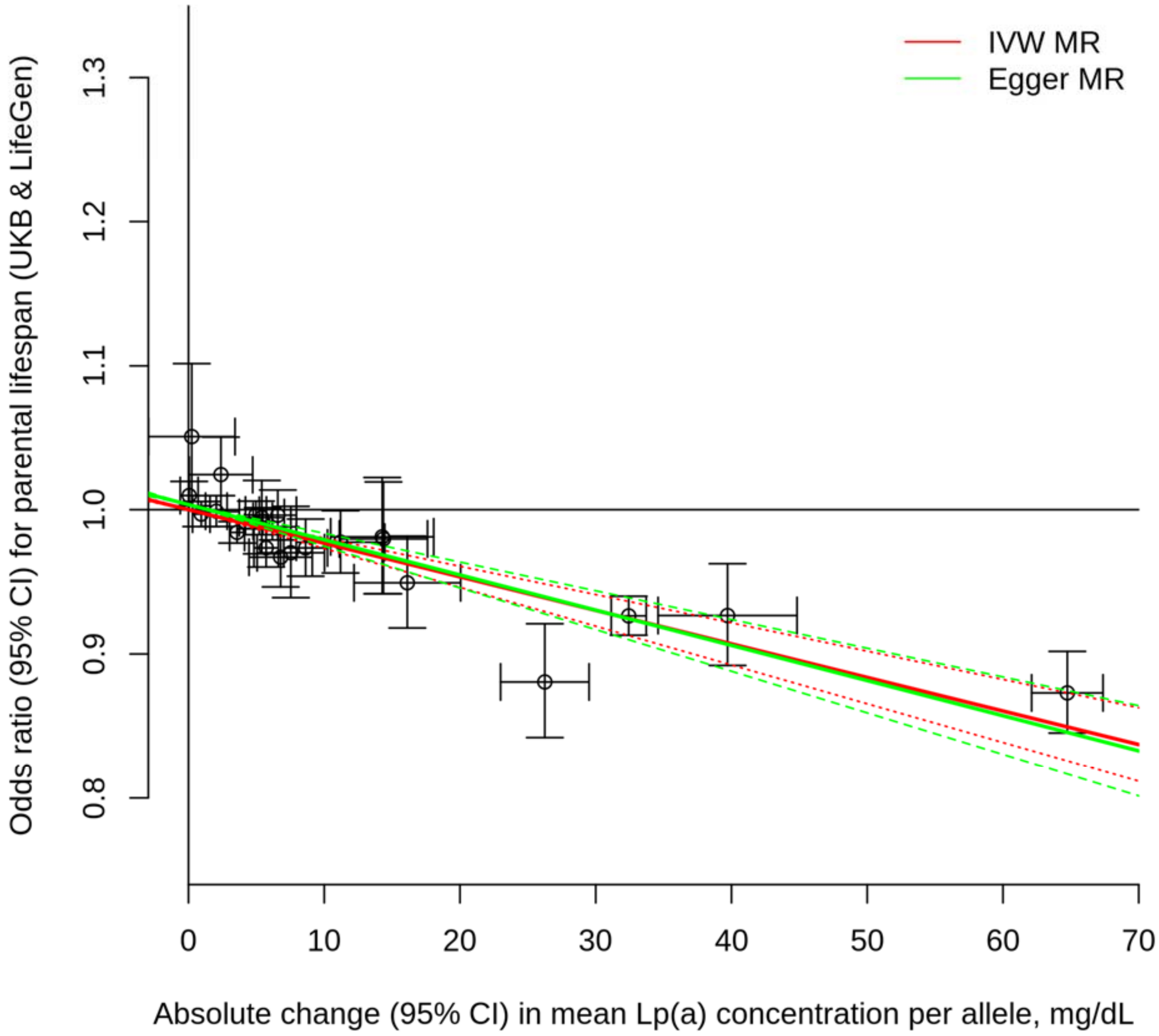

© 2020 Arsenault BJ et al. *JAMA Network Open.*

C)

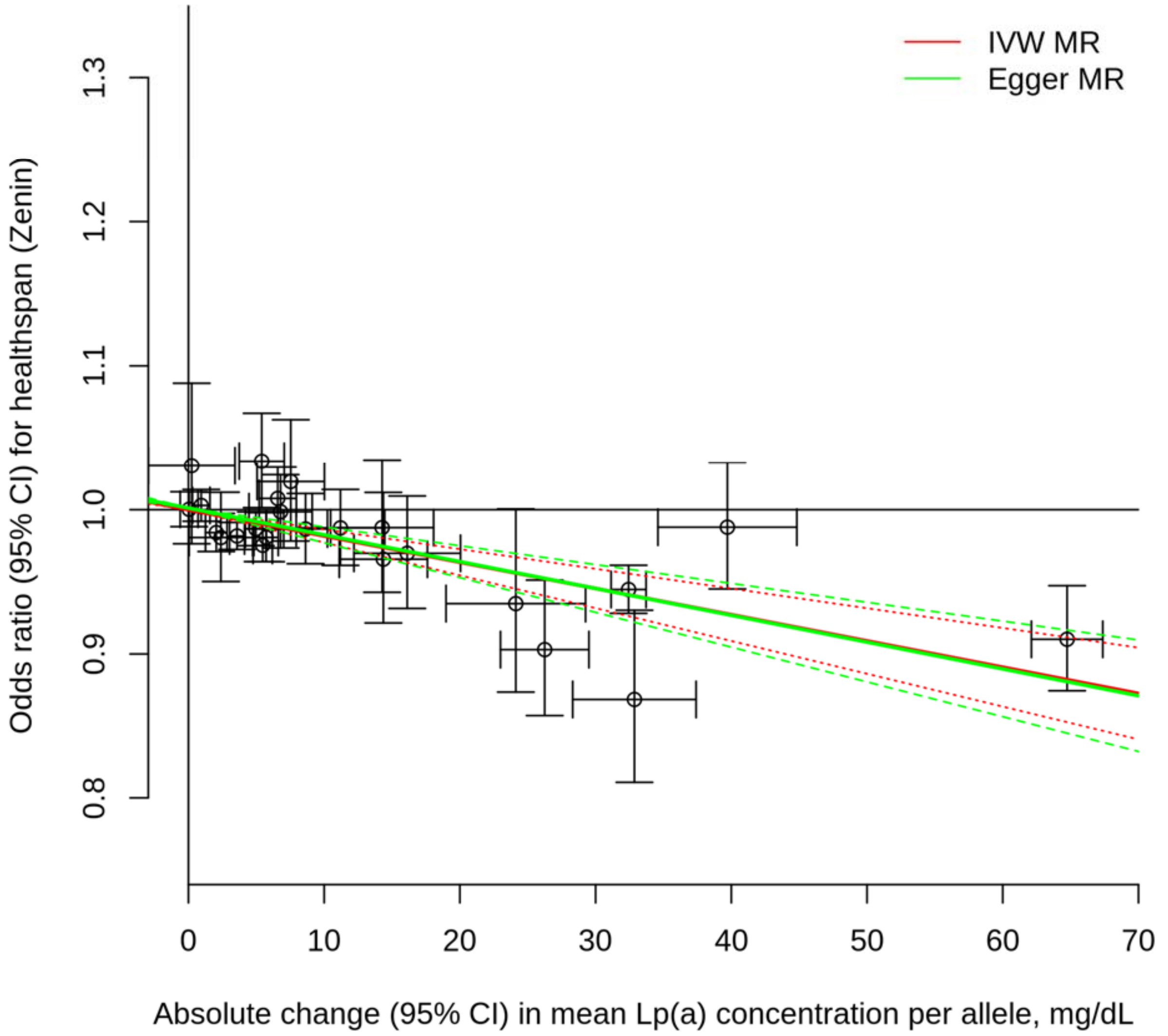

© 2020 Arsenault BJ et al. *JAMA Network Open.*

D)

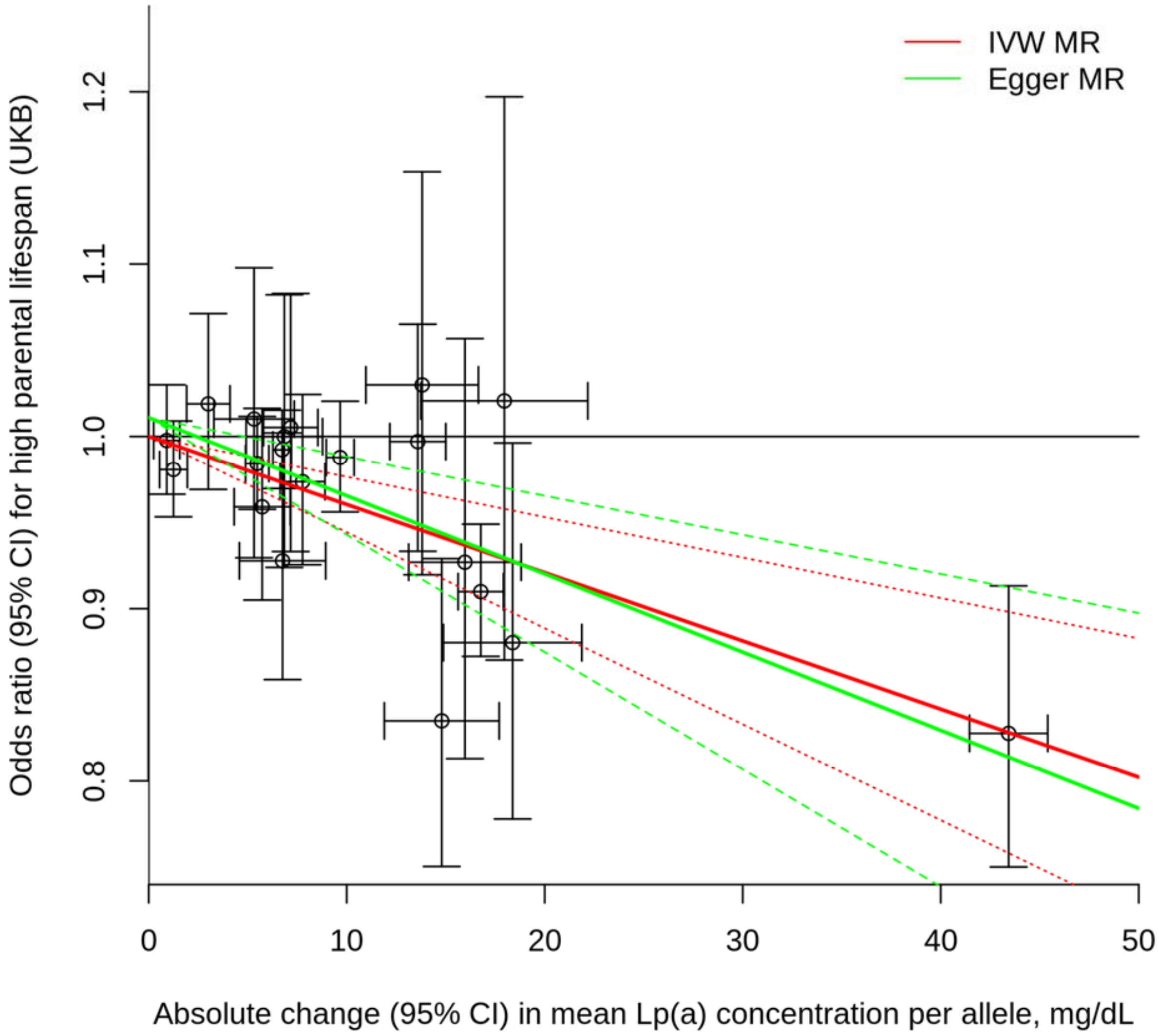

© 2020 Arsenault BJ et al. *JAMA Network Open.*

E)

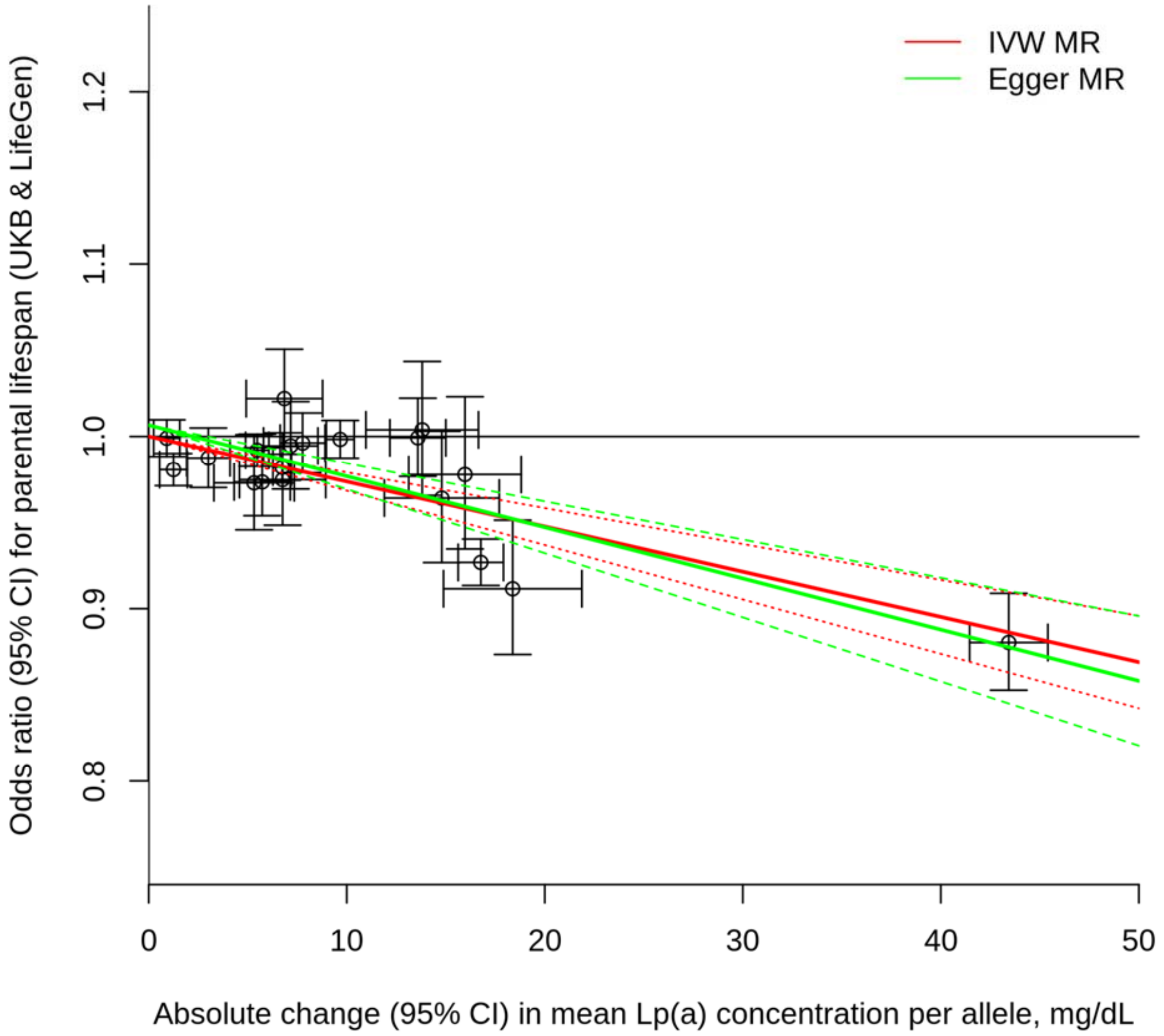

© 2020 Arsenault BJ et al. *JAMA Network Open.*

F)

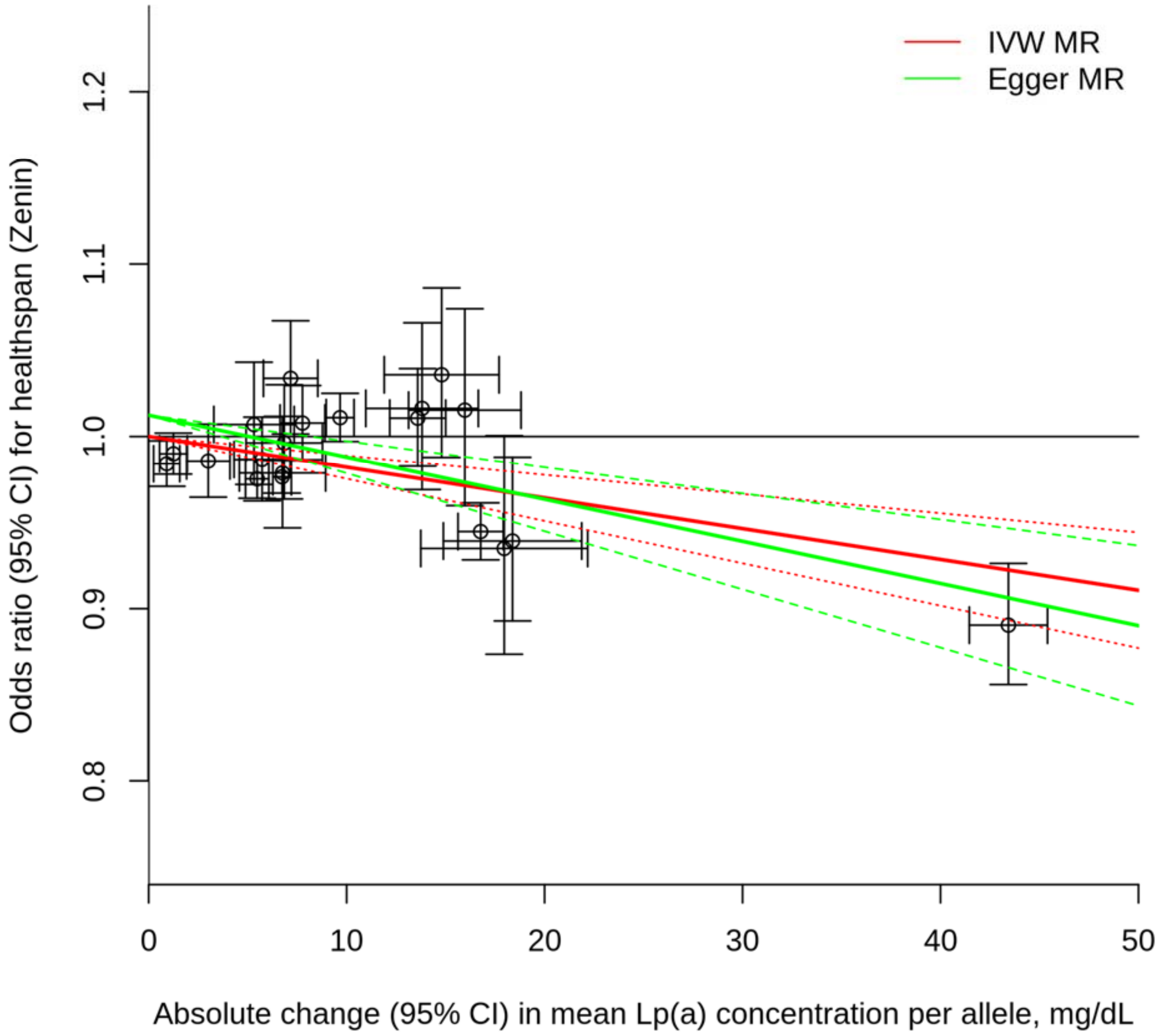

Association between SNPs at the *LPA* locus weighted for their impact on lipoprotein(a) levels from the study of Mack et al. (adjusted for age and sex) and higher parental lifespan in the UK Biobank (A), higher parental lifespan in the UK Biobank and LifeGen meta-analysis (B) higher healthspan in the UK Biobank (C). Also reported are the association between SNPs at the *LPA* locus weighted for their impact on lipoprotein(a) levels from the study of Mack et al. (adjusted for age, sex and apolipoprotein(a) isoform size) and higher parental lifespan in the UK Biobank (D), higher parental lifespan in the UK Biobank and LifeGen meta-analysis (E) higher healthspan in the UK Biobank (F). Each plotted point represents the effect of a single genetic variant on lipoprotein(a) levels (x-axis) and a high parental lifespan (y-axis). The red line represents the regression slope using the inverse-variance weighted method and the green line represents the regression slope using the Egger method. IVW-MR indicates inverse-variance weighted Mendelian randomization.

© 2020 Arsenault BJ et al. *JAMA Network Open.*

eFigure 6. Association between each lipoprotein(a)-raising variant (obtained from the study of Burgess et al.) and longevity phenotypes.
A)

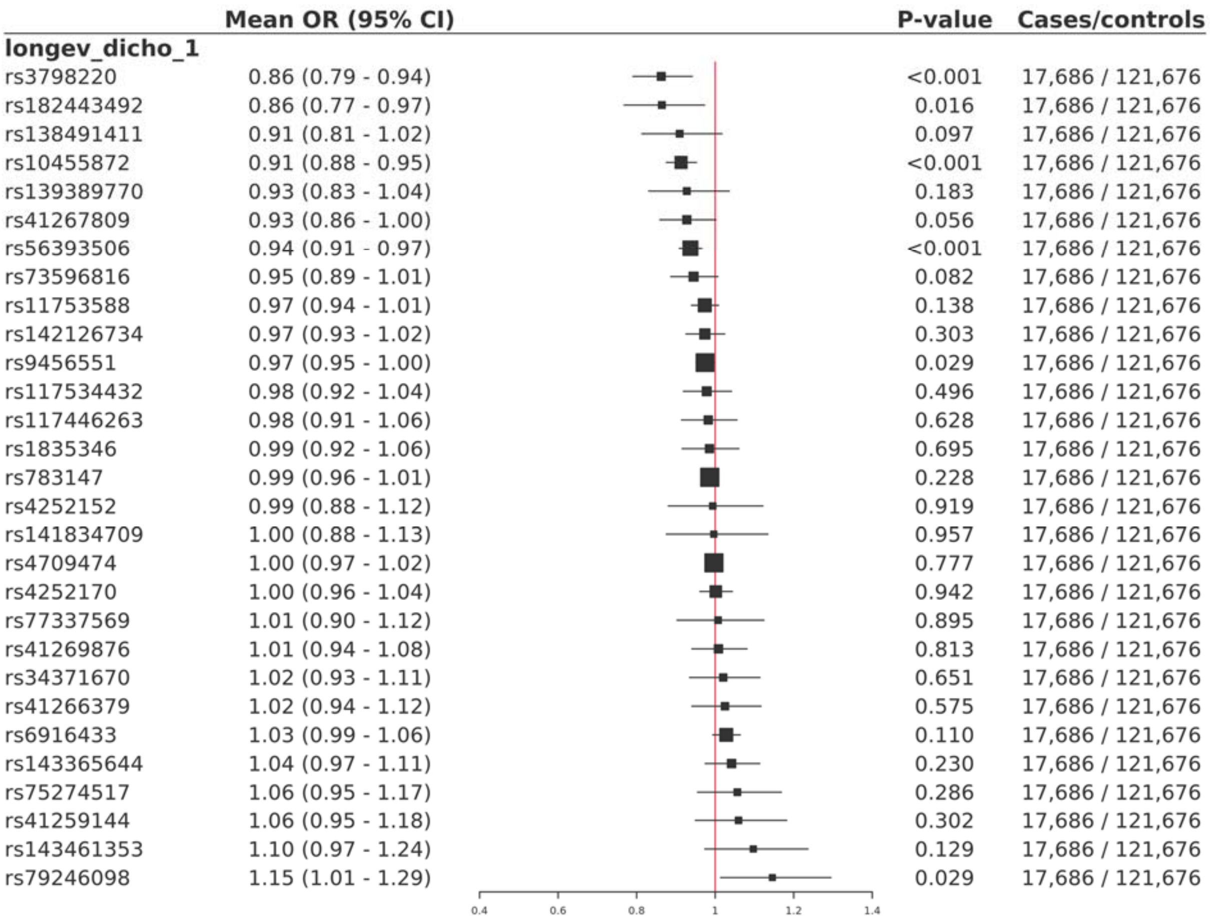

© 2020 Arsenault BJ et al. *JAMA Network Open.*

B)

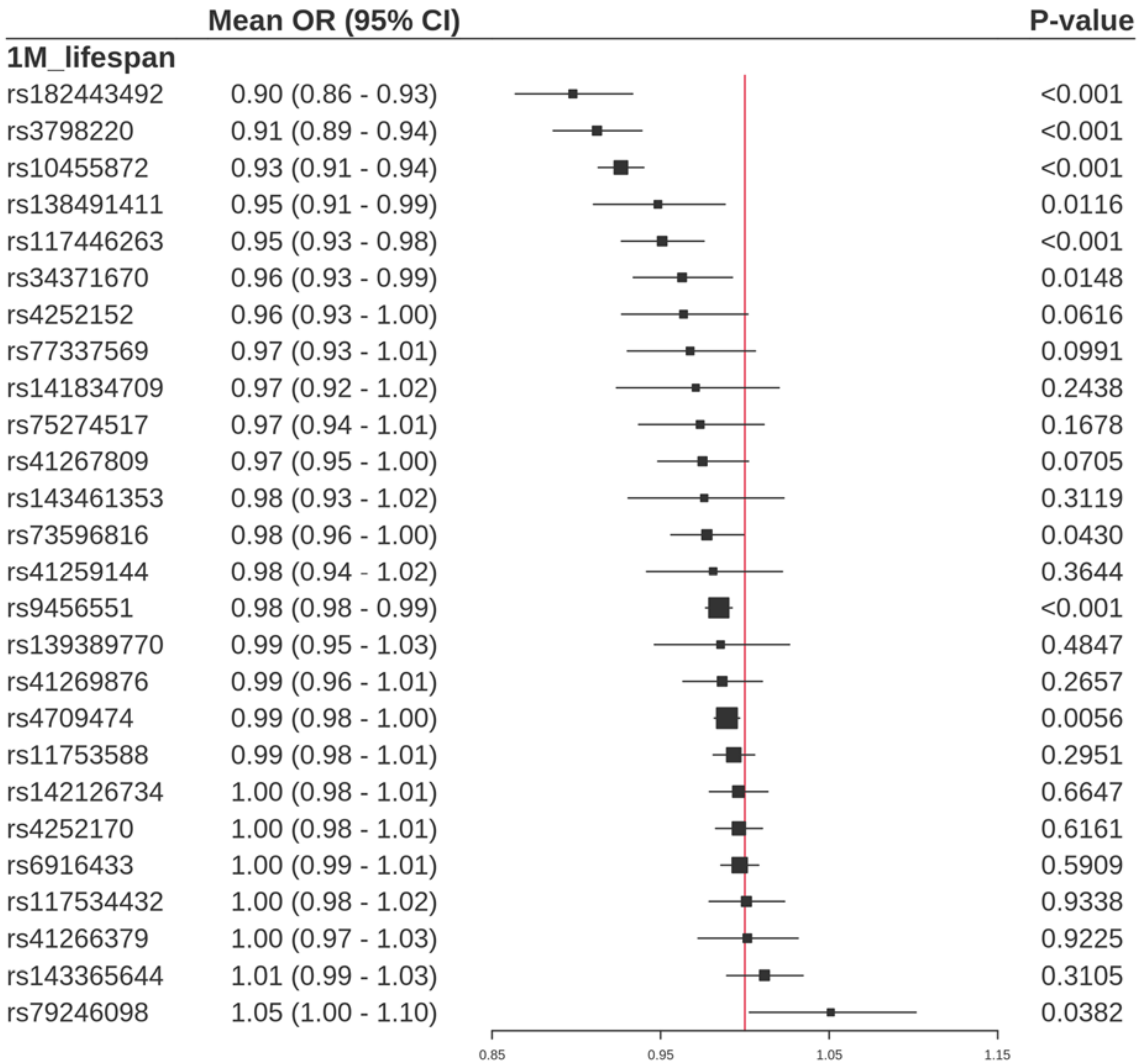

© 2020 Arsenault BJ et al. *JAMA Network Open.*

C)

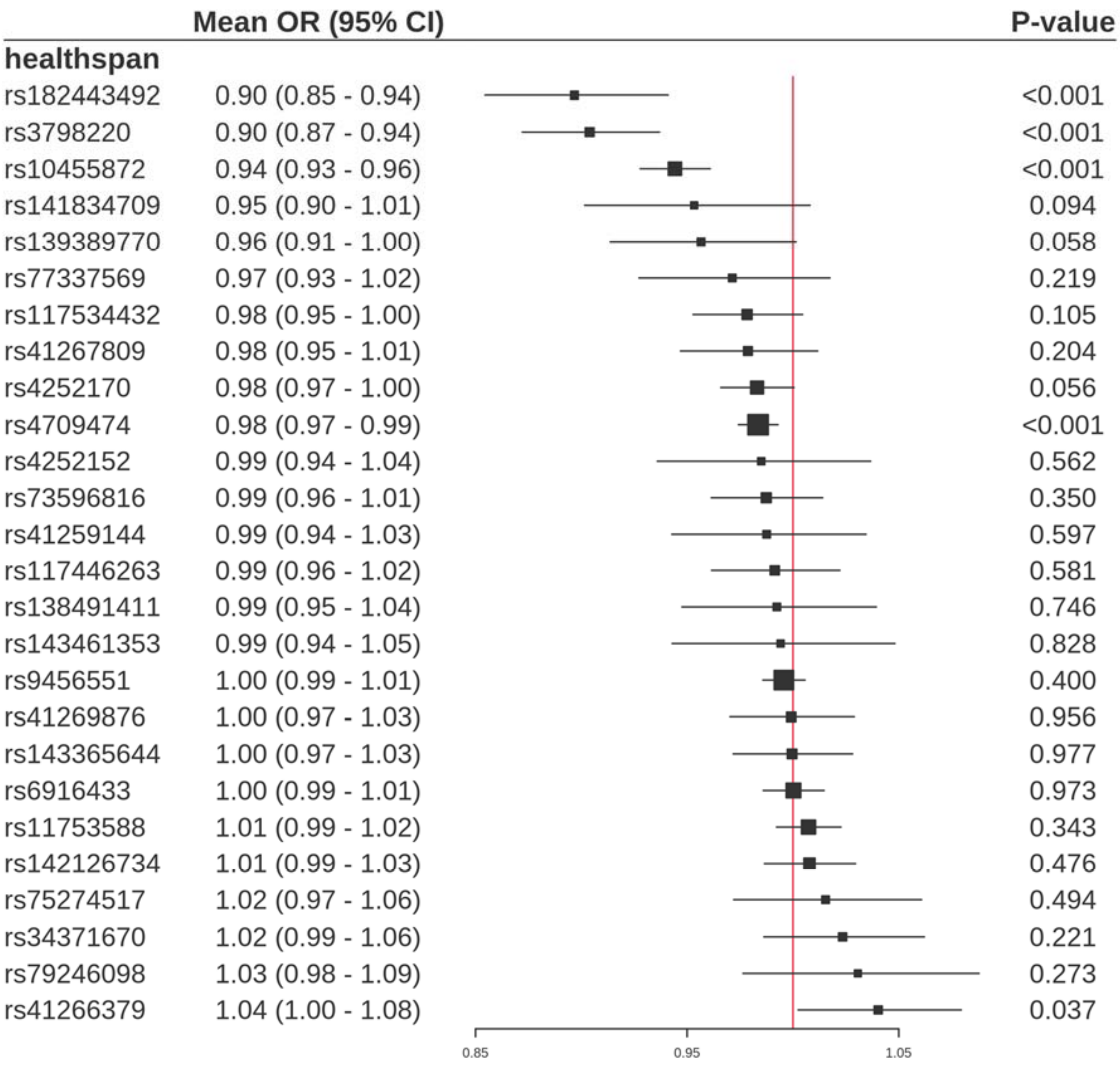

Results are presents for the following outcomes: Parental lifespan in the UK Biobank (A), parental lifespan in the UK Biobank and LifeGen meta-analysis and healthspan in the UK Biobank.

© 2020 Arsenault BJ et al. *JAMA Network Open.*

eFigure 7. Association between each lipoprotein(a)-raising variant (obtained from the study of Mack et al) without adjusting for apolipoprotein(a) isoform size and longevity phenotypes.

A)

	Mean OR (95% CI)	P-value	Cases/controls
**longev_dicho_1**			
rs186696265	0.84 (0.76 - 0.93)	<0.001	17,686 / 121,676
rs184278183	0.86 (0.75 - 0.98)	0.020	17,686 / 121,676
rs12204009	0.88 (0.74 - 1.04)	0.146	17,686 / 121,676
rs62441903	0.91 (0.82 - 1.02)	0.095	17,686 / 121,676
rs118039278	0.91 (0.87 - 0.95)	<0.001	17,686 / 121,676
rs143665477	0.94 (0.84 - 1.04)	0.218	17,686 / 121,676
rs73596816	0.95 (0.89 - 1.01)	0.082	17,686 / 121,676
rs41272114	0.96 (0.91 - 1.02)	0.158	17,686 / 121,676
rs142126734	0.97 (0.93 - 1.02)	0.303	17,686 / 121,676
rs6938647	0.98 (0.96 - 1.01)	0.256	17,686 / 121,676
rs520829	0.99 (0.97 - 1.01)	0.261	17,686 / 121,676
rs41269133	0.99 (0.95 - 1.03)	0.730	17,686 / 121,676
rs12529361	1.00 (0.97 - 1.03)	0.866	17,686 / 121,676
rs62440901	1.00 (0.97 - 1.03)	0.886	17,686 / 121,676
rs2140650	1.00 (0.97 - 1.02)	0.889	17,686 / 121,676
rs12664092	1.01 (0.93 - 1.08)	0.891	17,686 / 121,676
rs9457778	1.01 (0.98 - 1.04)	0.654	17,686 / 121,676
rs139699952	1.01 (0.95 - 1.07)	0.789	17,686 / 121,676
rs141463285	1.02 (0.87 - 1.20)	0.801	17,686 / 121,676
rs614754	1.03 (0.94 - 1.14)	0.470	17,686 / 121,676
rs76000021	1.04 (0.94 - 1.15)	0.459	17,686 / 121,676
rs143520616	1.04 (0.97 - 1.12)	0.301	17,686 / 121,676
rs41259144	1.06 (0.95 - 1.18)	0.302	17,686 / 121,676
rs79246098	1.15 (1.01 - 1.29)	0.029	17,686 / 121,676

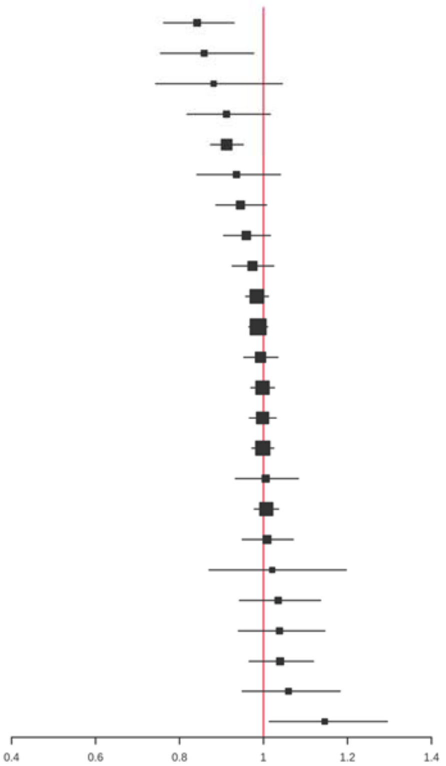

© 2020 Arsenault BJ et al. *JAMA Network Open.*

B)

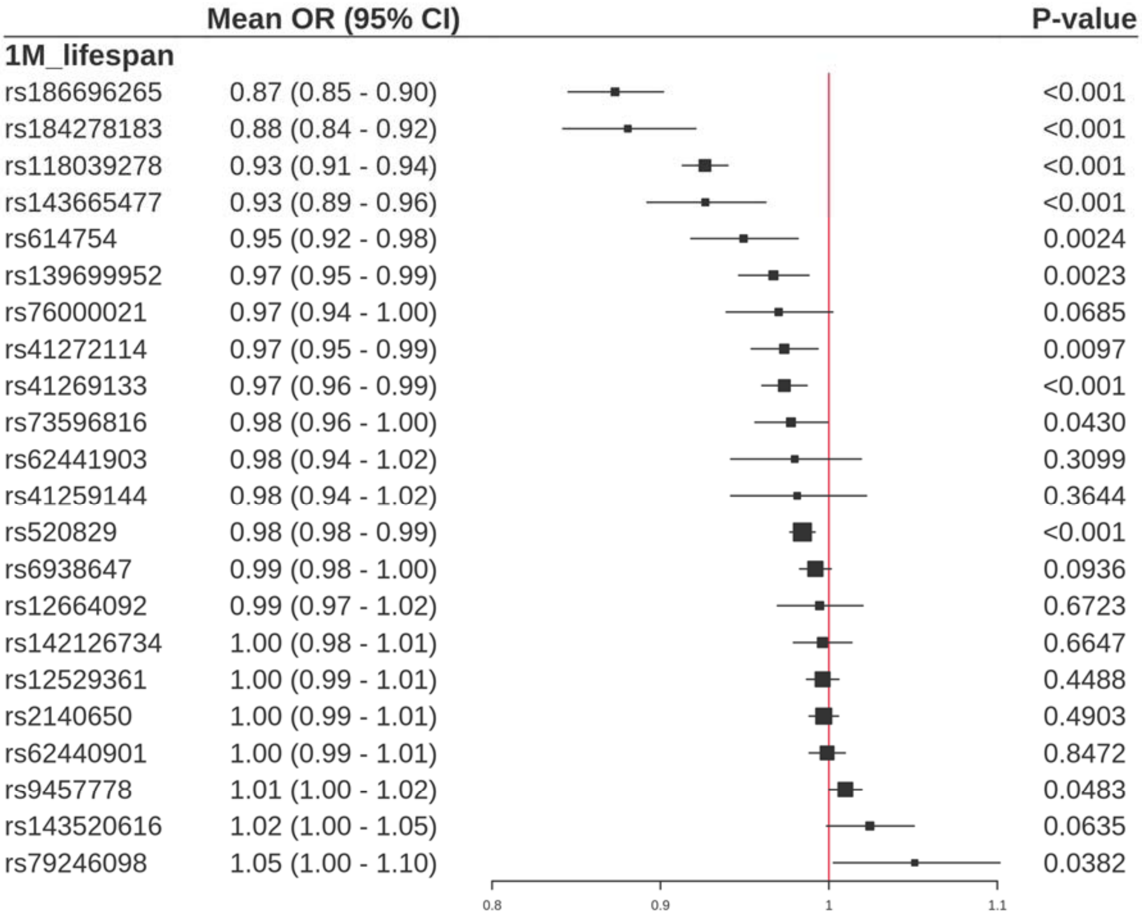

© 2020 Arsenault BJ et al. *JAMA Network Open.*

C)

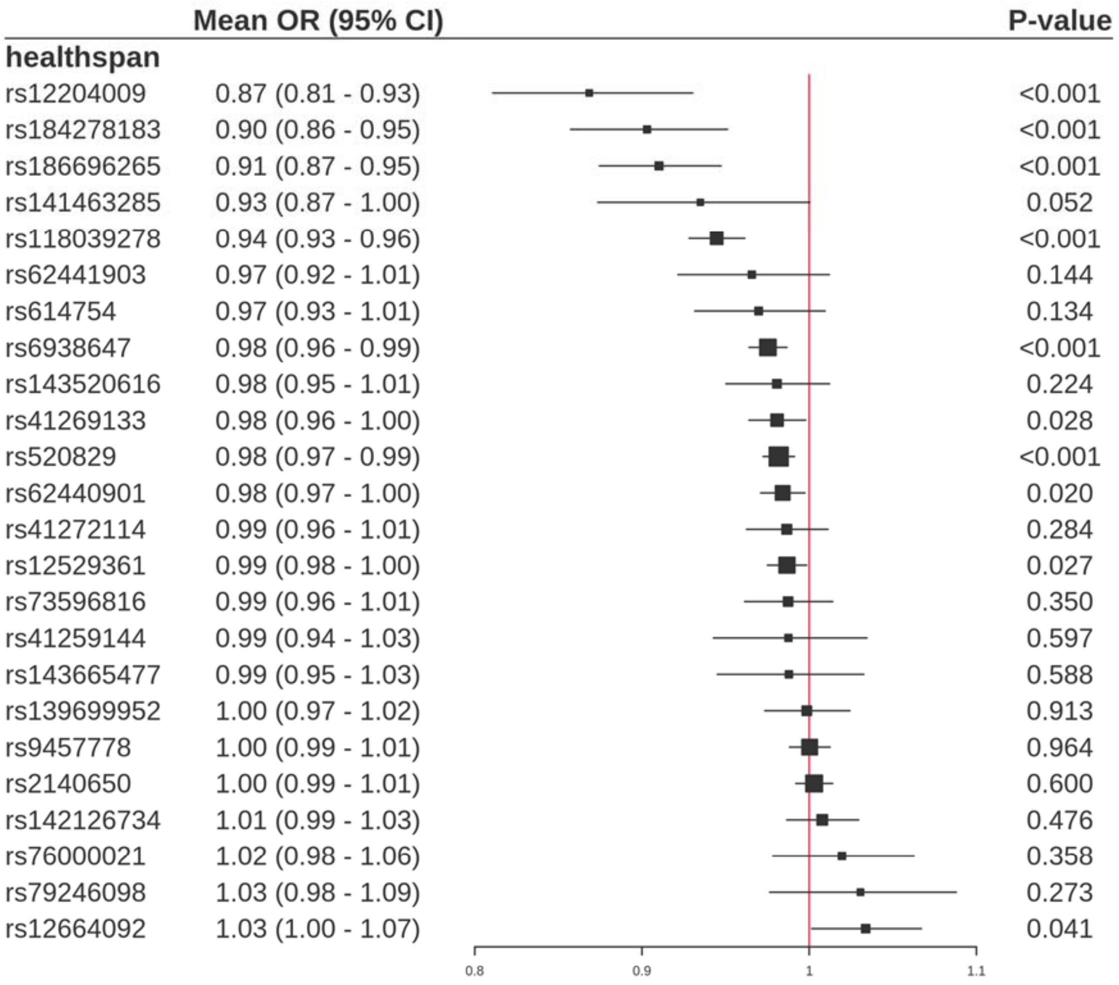

Results are presents for the following outcomes: Parental lifespan in the UK Biobank (A), parental lifespan in the UK Biobank and LifeGen meta-analysis and healthspan in the UK Biobank.

© 2020 Arsenault BJ et al. *JAMA Network Open.*

eFigure 8. Association between each lipoprotein(a)-raising variant (obtained from the study of Mack et al) after adjusting for apolipoprotein(a) isoform size and longevity phenotypes.

A)

	Mean OR (95% CI)	P-value	Cases/controls
**longev_dicho_1**			
rs140570886	0.83 (0.75 - 0.91)	<0.001	17,686 / 121,676
rs12207325	0.83 (0.75 - 0.93)	<0.001	17,686 / 121,676
rs147010904	0.88 (0.78 - 1.00)	0.043	17,686 / 121,676
rs55730499	0.91 (0.87 - 0.95)	<0.001	17,686 / 121,676
rs145470851	0.93 (0.81 - 1.06)	0.257	17,686 / 121,676
rs41267809	0.93 (0.86 - 1.00)	0.056	17,686 / 121,676
rs41272114	0.96 (0.91 - 1.02)	0.158	17,686 / 121,676
rs142126734	0.97 (0.93 - 1.02)	0.303	17,686 / 121,676
rs3798221	0.98 (0.95 - 1.01)	0.179	17,686 / 121,676
rs6938647	0.98 (0.96 - 1.01)	0.256	17,686 / 121,676
rs75692336	0.99 (0.96 - 1.02)	0.459	17,686 / 121,676
rs7769879	0.99 (0.97 - 1.02)	0.506	17,686 / 121,676
rs75234242	1.00 (0.93 - 1.07)	0.930	17,686 / 121,676
rs62440901	1.00 (0.97 - 1.03)	0.886	17,686 / 121,676
rs112842440	1.00 (0.92 - 1.08)	0.999	17,686 / 121,676
rs12664092	1.01 (0.93 - 1.08)	0.891	17,686 / 121,676
rs41267807	1.01 (0.93 - 1.10)	0.812	17,686 / 121,676
rs9295143	1.02 (0.97 - 1.07)	0.463	17,686 / 121,676
rs141463285	1.02 (0.87 - 1.20)	0.801	17,686 / 121,676
rs117026595	1.03 (0.92 - 1.15)	0.610	17,686 / 121,676

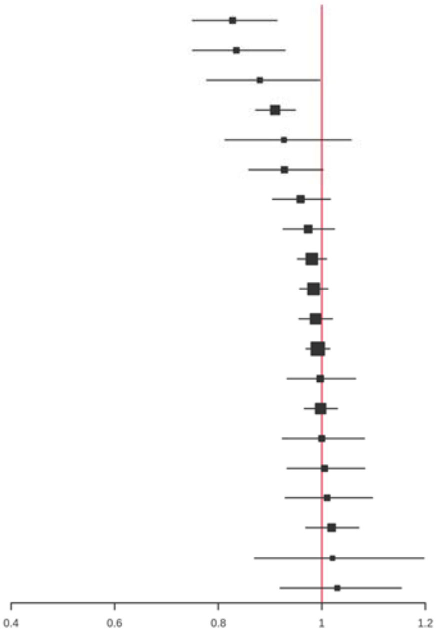

© 2020 Arsenault BJ et al. *JAMA Network Open.*

B)

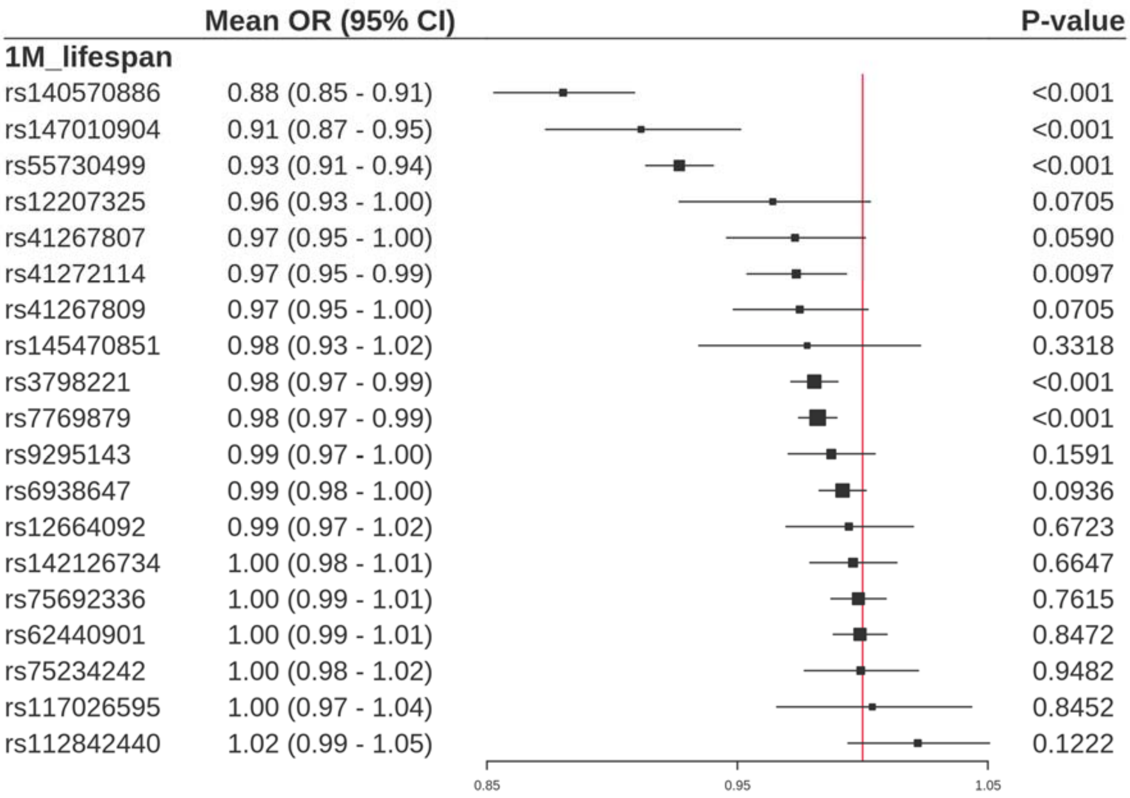

© 2020 Arsenault BJ et al. *JAMA Network Open.*

C)

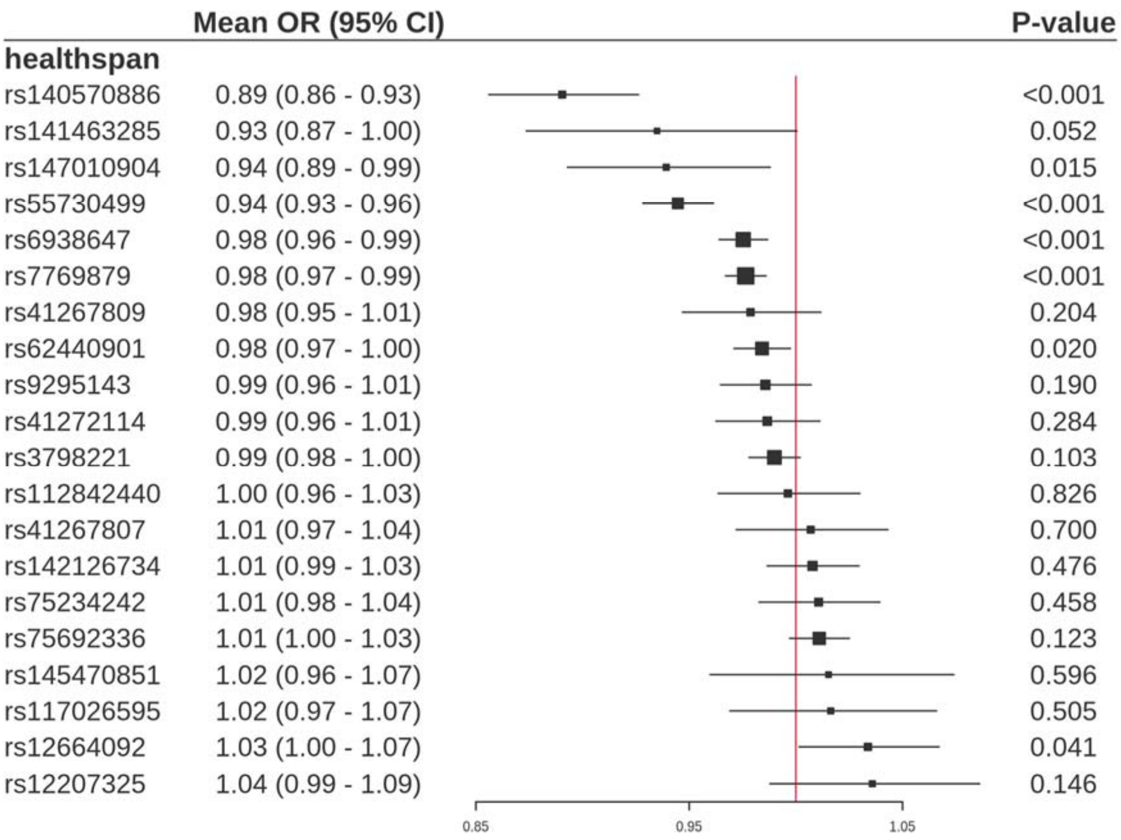

Results are presents for the following outcomes: Parental lifespan in the UK Biobank (A), parental lifespan in the UK Biobank and LifeGen meta-analysis and healthspan in the UK Biobank.

© 2020 Arsenault BJ et al. *JAMA Network Open.*

eFigure 9. Health hazards associated with high lipoprotein(a) levels in the EPIC-Norfolk study by baseline age categories.

A)

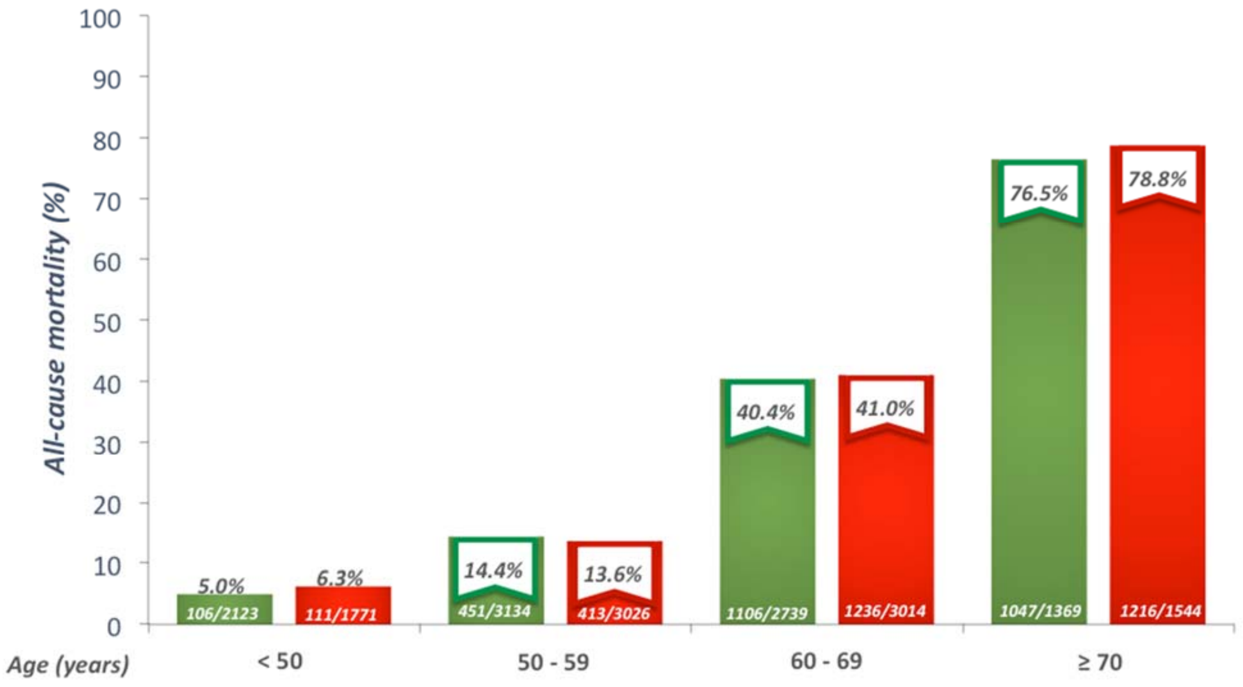

B)

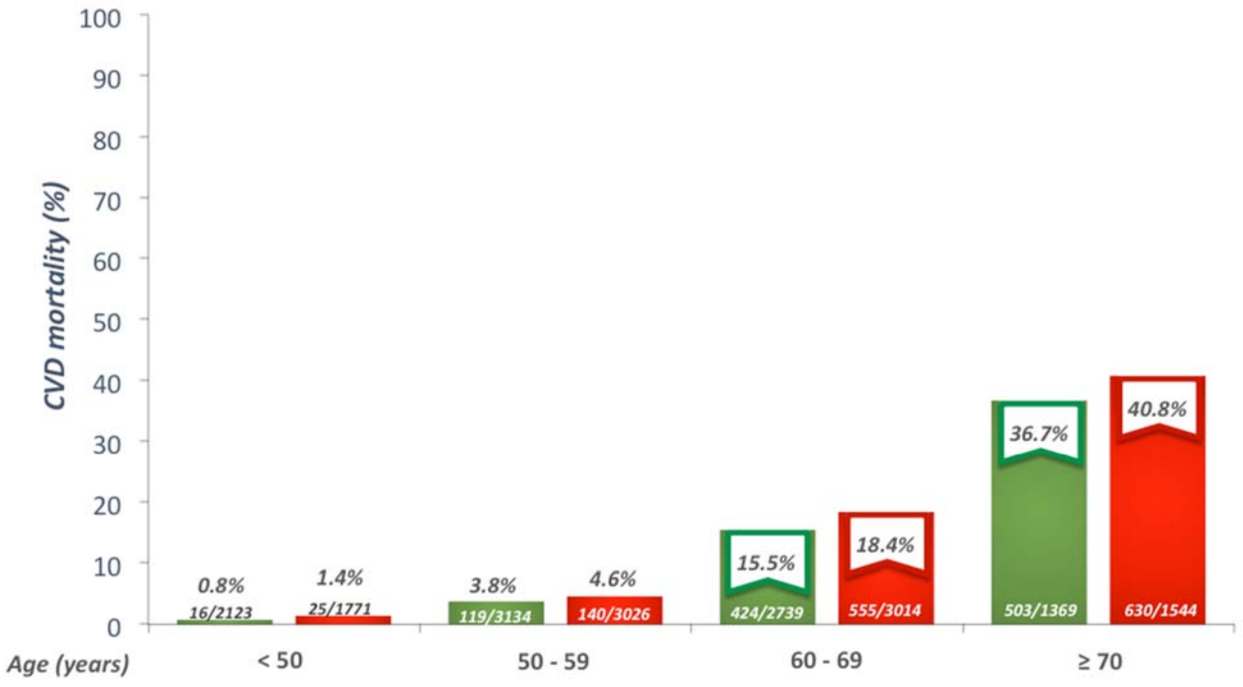

© 2020 Arsenault BJ et al. *JAMA Network Open.*

C)

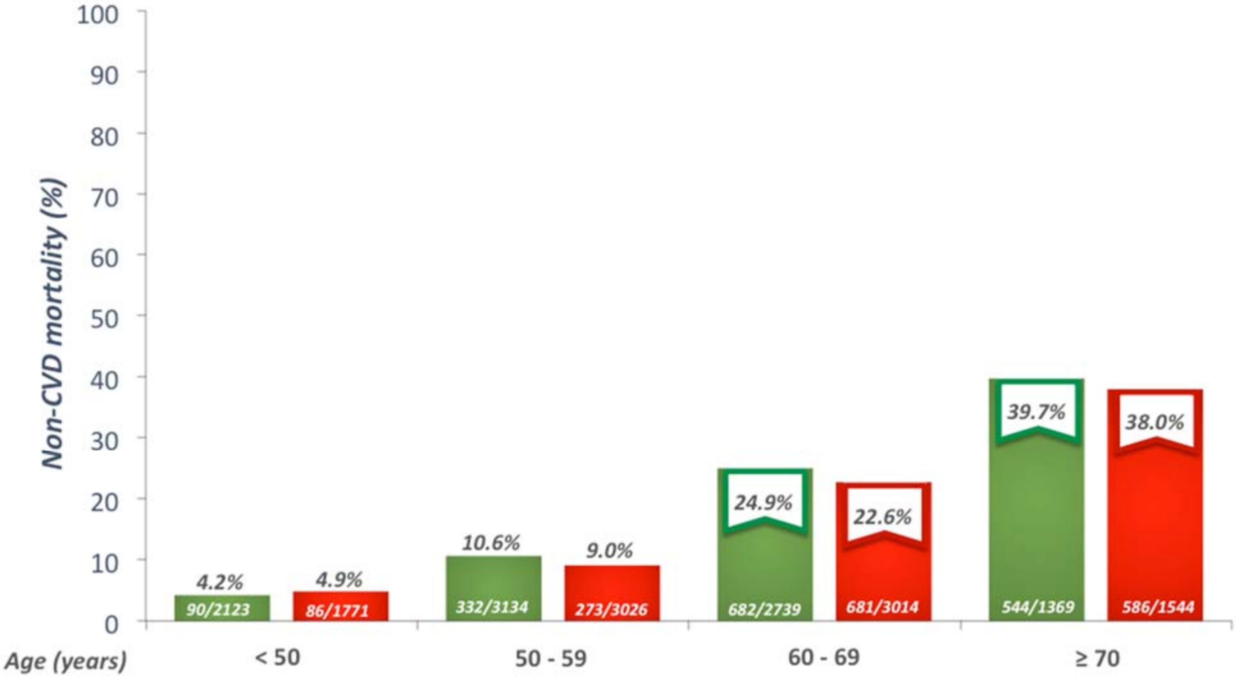

D)

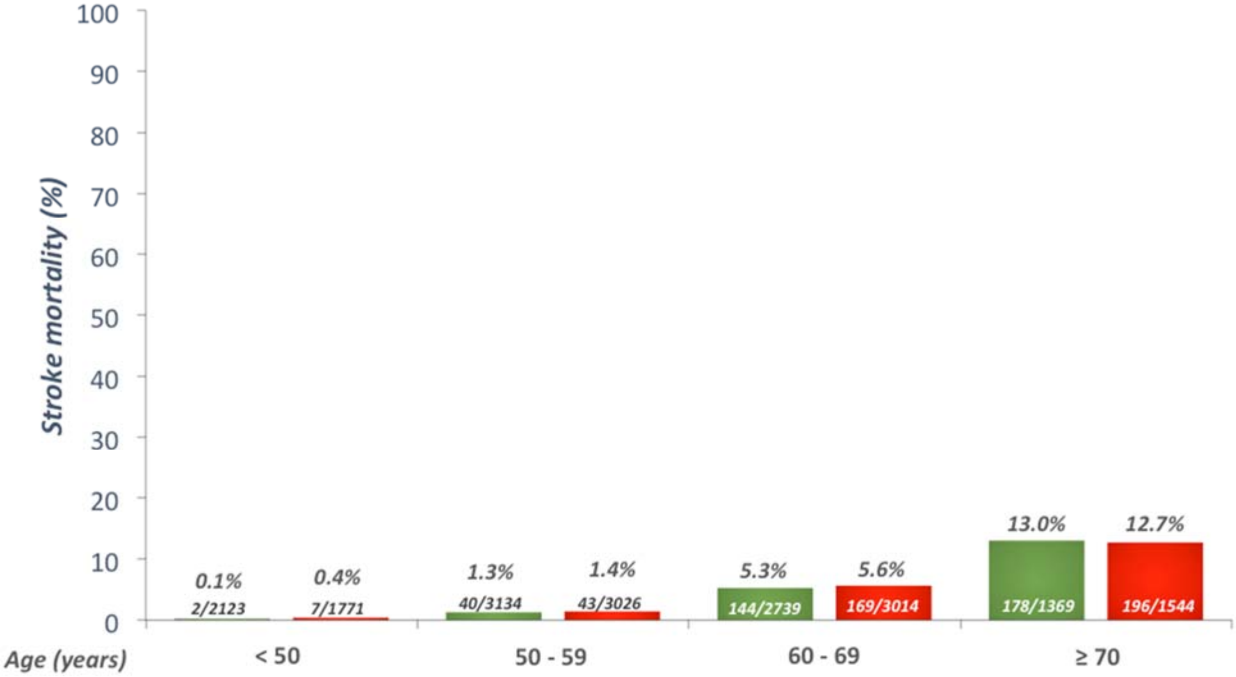

© 2020 Arsenault BJ et al. *JAMA Network Open.*

E)

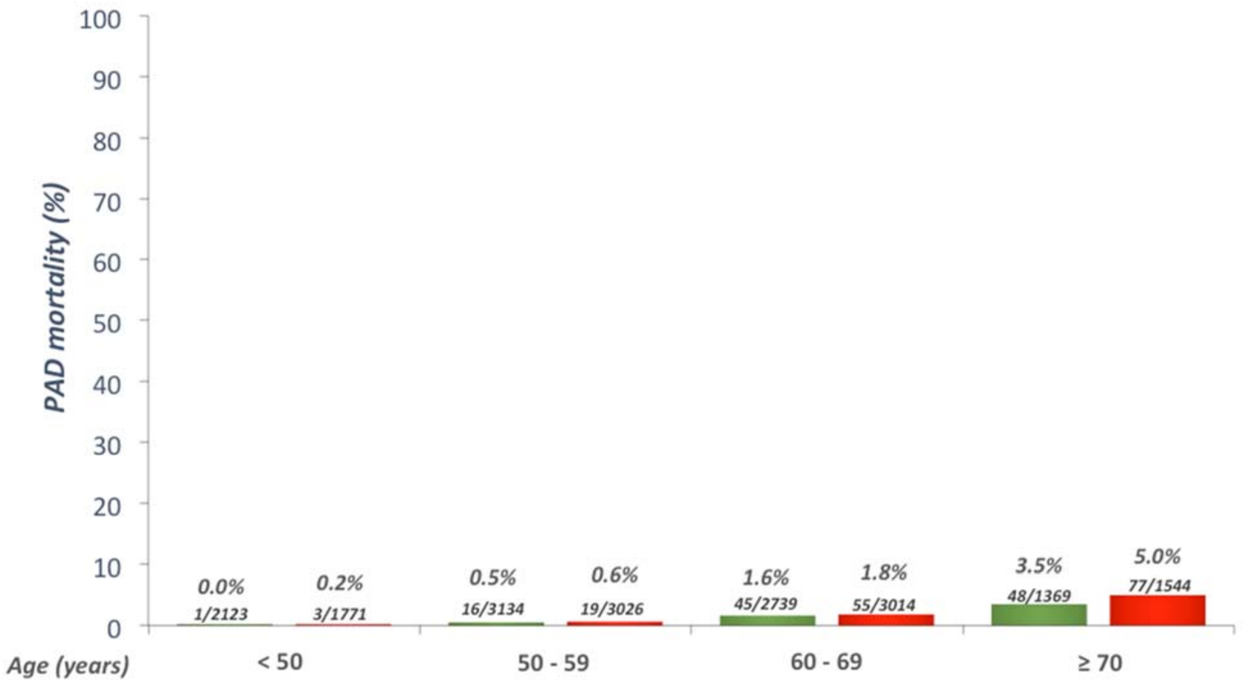

F)

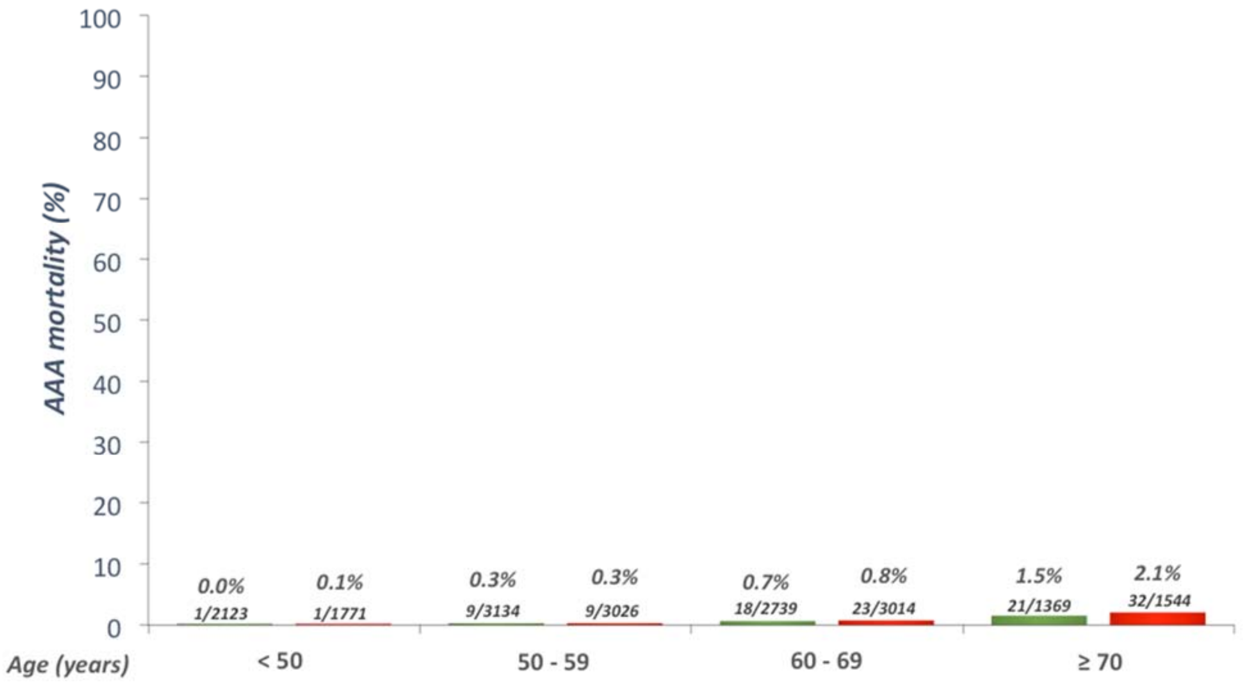

The figure present event rates for A) all-cause mortality B) cardiovascular disease (CVD) mortality, C) non-CVD mortality, D) stroke mortality, E) peripheral artery disease (PAD) mortality and F) abdominal aortic aneurism (AAA) mortality in participants of the EPIC-Norfolk study with high (≥50 mg/dL, red) or low (<50 mg/dL, green) lipoprotein(a) levels by baseline age categories

© 2020 Arsenault BJ et al. *JAMA Network Open.*

eFigure 10. Impact of lipoprotein(a) levels on non-cardiovascular disease mortality in the EPIC-Norfolk.

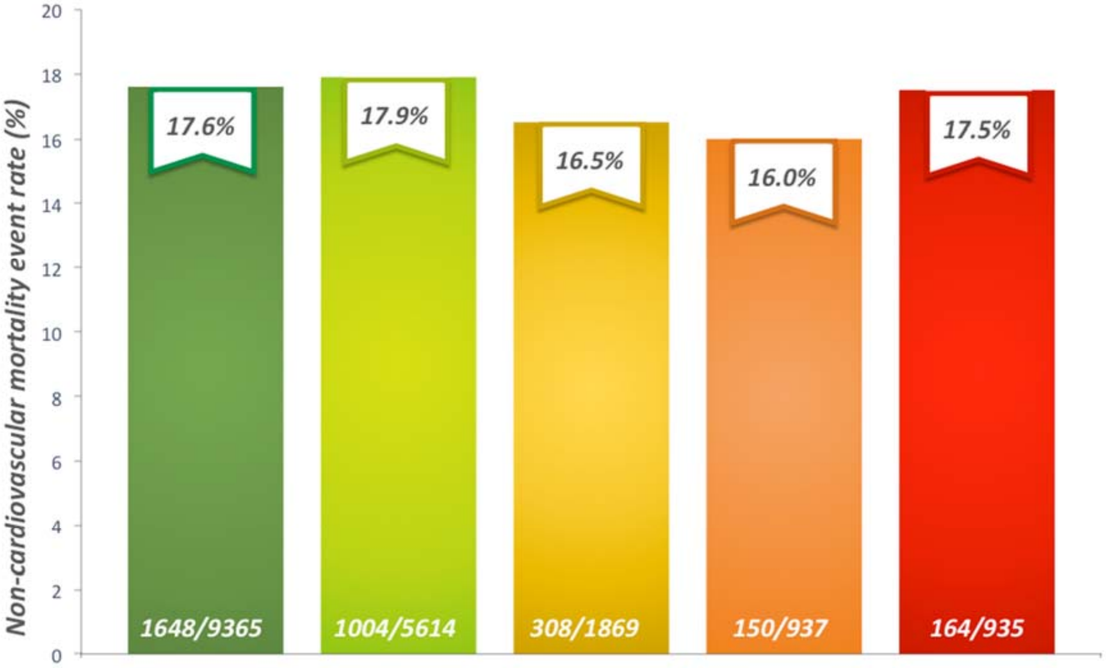

Percentiles	< 50	50-80	81-90	91-95	≥ 95
Lp(a) range (mg/dL)	≤ 11.4	11.4-35.0	35.0-53.3	53.3-69.7	> 69.7
Model 1	1.00	0.91 (0.84-0.98)	0.92 (0.81-1.04)	0.88 (0.74-1.04)	0.94 (0.80-1.10)
Model 2	1.00	0.91 (0.84-0.98)	0.94 (0.83-1.06)	0.88 (0.74-1.04)	0.93 (0.79-1.10)

The figure present event rates and hazard ratios for non-cardiovascular mortality in participants of the EPIC-Norfolk study by extreme high lipoprotein(a) levels. Model 1 is adjusted for age and sex. Model 2 is adjusted for age, sex, smoking, body mass index, systolic blood pressure, diabetes mellitus and estimated glomerular filtration rate.

© 2020 Arsenault BJ et al. *JAMA Network Open.*

eFigure 11. Event rates and hazard ratios for all-cause (A) and cardiovascular mortality (B) in participants of the EPIC-Norfolk study by number of lipoprotein(a)-raising alleles.

A)

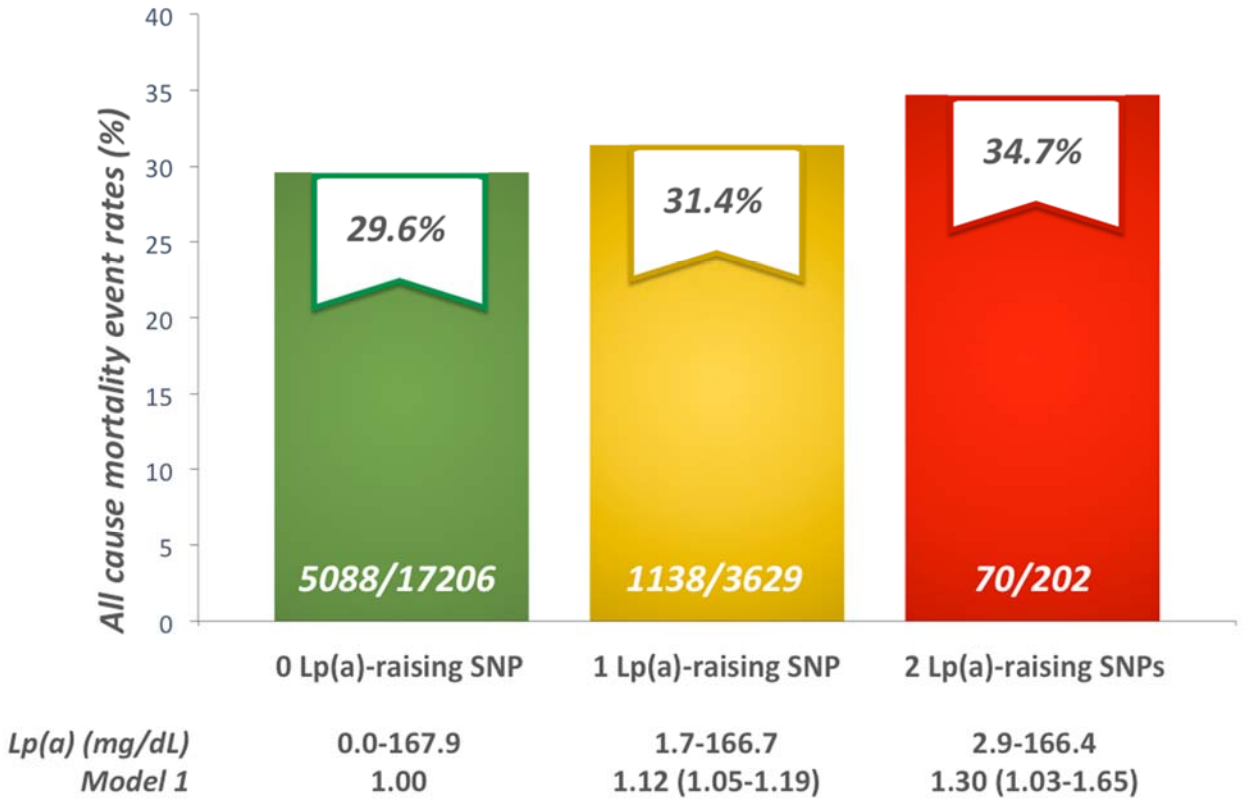

© 2020 Arsenault BJ et al. *JAMA Network Open.*

B)

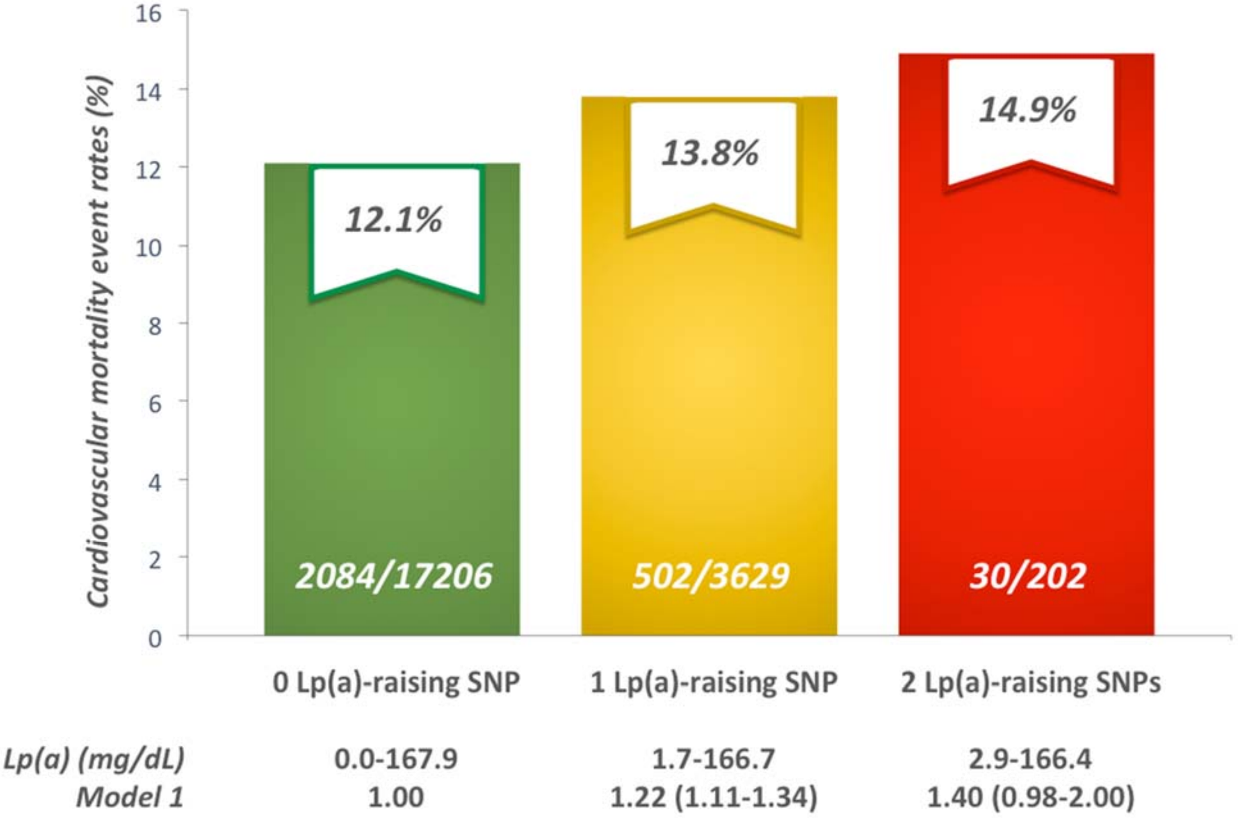

Model 1 is adjusted for age and sex.

© 2020 Arsenault BJ et al. *JAMA Network Open.*
